# Investigating the Impact That Diagnostic Screening with Lateral Flow Devices Had on the Rabies Surveillance Program in Zanzibar, Tanzania

**DOI:** 10.3390/microorganisms12071314

**Published:** 2024-06-27

**Authors:** Ali Z. Moh’d, Andre Coetzer, Ayla J. Malan, Terence P. Scott, Ramadhan J. Ramadhan, Nicolette Wright, Louis H. Nel

**Affiliations:** 1 Department of Livestock Development, Ministry of Agriculture, Irrigation, Natural Resources and Livestock, Zanzibar P.O. Box 159, Tanzania; 2 Global Alliance for Rabies Control, Manhattan, KS 66502, USA; andre.coetzer@rabiesalliance.org (A.C.); terence.scott@rabiesalliance.org (T.P.S.); 3 Department of Biochemistry, Genetics and Microbiology, Faculty of Natural and Agricultural Sciences, University of Pretoria, Pretoria 0002, South Africa

**Keywords:** rabies, lateral flow devices, diagnosis, surveillance, rapid test kits, in-field testing

## Abstract

With the global impetus for the elimination of canine-mediated human rabies, the need for robust rabies surveillance systems has become ever more important. Many countries are working to improve their rabies surveillance programs and, as a result, the reported use of lateral flow devices (LFDs) is increasing. Despite their known diagnostic limitations, previous studies have hypothesised that the benefits associated with LFDs could make them potentially quite useful towards improving the overall robustness of surveillance programs. To test this, a best practice standard operating procedure was developed which was used to guide the implementation of the ADTEC LFD as a diagnostic screening tool in Zanzibar. Over the course of the first 22 months of this investigation, 83 samples were subjected to in-field diagnostic screening, coupled with subsequent laboratory confirmation, and only one false-negative result was detected. Furthermore, the findings of our investigation indicated that the routine use of LFDs as a diagnostic screening tool resulted in a four-fold increase in the number of samples subjected to rabies diagnosis per month and a three-fold increase in the number of wards where samples were collected per year. Our findings suggest that LFDs could play a noteworthy role in improving the robustness of surveillance systems by increasing the number of samples tested and promoting diagnostic screening in areas distant from laboratories. Their implementation would, however, need to be carefully controlled through standardised protocols that align with the international best practices to ensure their judicious use.

## 1. Introduction

Despite being a vaccine-preventable disease, canine-mediated rabies still poses a significant burden on public health systems in up to 150 countries—the majority of which are in Africa and Asia—and results in the death of tens of thousands of people each year [1,2]. In line with the Global Strategic Plan to end human deaths from dog-mediated rabies by 2030 (Zero by 30), governments need to focus their efforts on strengthening surveillance programs that often lack the funding required for their adequate implementation so that progress towards disease elimination can be made [1,3]. By strengthening rabies surveillance networks, countries could narrow the gap between predicted and reported data, giving a true representation of the threat that dog-mediated rabies poses to a country’s public health system [4,5,6]. With this evidence base, rabies can be prioritised, and the ‘cycle of neglect’ can be broken—contributing greatly towards the eventual control and elimination of the disease [5,7]. In addition, grassroots-level data could be used to direct disease intervention campaigns whereby valuable resources are allocated towards identified high-risk areas [8]. This approach not only ensures that the available resources are utilised optimally, but it also ensures the sustainability of control and elimination programs by minimising their required operational resources [8,9].

For the gathering of disease burden data, resource-limited rabies-endemic countries typically utilise passive surveillance systems that rely on the evaluation and testing of suspected rabid animals involved in human exposures. This approach is often limited to geographical areas with high human populations and established public health infrastructures, which largely limits their effectiveness to urban and peri-urban settings [10]. Considering that rabies primarily affects rural and underserved populations, passive surveillance systems result in many suspected rabies cases going undetected, leading to an underestimation of the true disease burden [4,7]. In contrast, more robust active surveillance systems rely on a targeted sampling approach focusing on animals that (i) exhibit abnormal behaviour, (ii) are found dead, or (iii) have been killed by vehicles [10]. While active surveillance programs result in more samples being tested—placing greater strain on the surveillance network—they offer significant advantages by providing additional and more diverse epidemiological data not typically provided by a passive surveillance system [10,11]. 

While rabies-endemic countries should ideally strive towards the implementation of both passive and active surveillance programs, the increased number of samples tends to highlight the most significant barriers hindering surveillance in low- and middle-income countries. These include the inability to deliver samples to the nearest diagnostic facility because of limited human and financial resources, poor infrastructure, and the inability to maintain a cold chain during transportation [6,12,13]. One solution to alleviate these difficulties would be to decentralise rabies diagnosis to limit the distance that samples need to be transported before they reach a laboratory that can implement a diagnostic assay recommended by the World Organization for Animal Health (WOAH) [6,10]. Nevertheless, this approach still requires significant financial investment and sustained funding to establish the diagnostic assay, maintain the required laboratory infrastructure, and operationalise animal health professionals at the laboratory and field levels [14,15,16]. 

Due to their efficiency, specificity, and reliability, diagnostic laboratories should always utilise WOAH-recommended diagnostic assays for the confirmation of clinical cases to aid with decision making (e.g., the continuation of human rabies post-exposure prophylaxis), determining the general disease prevalence, and official disease reporting [17]. However, rabies surveillance programs could be supplemented with diagnostic tests that are not recommended by WOAH, permitting that they are used to improve the general disease surveillance by acting as a diagnostic screening mechanism and that they are not used in the place of WOAH-recommended diagnostic assays for any official purposes [10,11]. Therefore, these supplementary diagnostic tests could be used in conjunction with WOAH-recommended diagnostic assays to improve or enhance surveillance capacities.

One such test that could enable the diagnostic screening of suspected rabies cases—without the need for significant infrastructural changes—is an immunochromatographic lateral flow device (LFD), which allows for the rapid detection of rabies both inside and outside of laboratory settings. Compared to the WOAH-recommended diagnostic assays, LFDs also offer significant benefits in terms of ease-of-use, cost-effectiveness, and speed [10,12,18,19,20]. Despite these benefits, it is well documented that LFDs suffer from a reduced diagnostic sensitivity due to their inability to detect viral antigens at low concentrations [12,18,20,21]. Regardless of the shortcomings associated with many of the commercially available LFDs and the fact that they are not a WOAH-recommended diagnostic assay [17], many rabies-endemic countries continue to utilise them in both the field [22,23,24,25,26,27,28,29] and laboratory settings [19,27,30,31,32,33,34]. As the use of LFDs evidently cannot be avoided, it is vital that their implementation is managed in such a way that they can be used effectively to improve surveillance efforts while their shortcomings are taken into consideration. 

Unguja island (more commonly known as Zanzibar) is a semi-autonomous region of the United Republic of Tanzania that has been endemic for dog-mediated rabies since the 1990s, despite the ongoing efforts of the Revolutionary Government of Zanzibar to eliminate the disease [8,35,36]. To quantify the burden of rabies and aid the government in eliminating the disease by using a strategic disease intervention approach, a rabies surveillance system was established across the island by implementing the direct, rapid immunohistochemical test (DRIT) assay at the Zanzibar Central Veterinary Laboratory (ZCVL) in 2016 [8]. This passive surveillance system (which has been operational since its introduction) remains functional, but additional resolution is required. The aim of this investigation was, thus, to determine the benefits of an active rabies surveillance system using LFDs as a diagnostic screening tool provided for Zanzibar’s rabies surveillance capacity. To this end, a best practice standard operating procedure (SOP) was developed and used to guide the implementation of a commercially supplied ‘Rabies Ag test’ LFD (ADTEC Co., Ltd., Oita, Japan)—hereafter referred to as the ADTEC LFD—as a diagnostic screening tool across the island. In this investigation, we evaluated the diagnostic efficacy of the ADTEC LFDs and determined the impact that their routine use had on improving the frequency of sample submissions and the geographical distribution of collected samples.

## 2. Materials and Methods

### 2.1. Ethical Permission

No ethical approval was necessary for the implementation of the rabies surveillance activities, as samples were collected by the Department of Livestock Development, who are mandated to undertake routine animal rabies surveillance across the island. Furthermore, animal health professionals are authorised by law to humanely euthanise and collect samples from any animals suspected of having a rabies diagnosis through Zanzibar’s Animal Resource Act, Number 11 of 1999. The Faculty of Natural and Agricultural Sciences (University of Pretoria) approved the retrospective analysis of the data used in this study (Approval number: NAS176/2023).

### 2.2. Project Site

**Figure 1:**
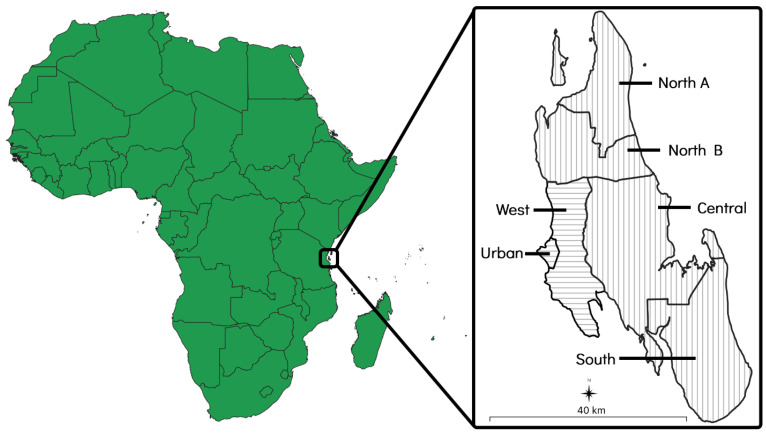
Map of Zanzibar showing its location and size in relation to mainland Africa. The two primarily urban districts have been indicated with horizontal lines and the four primarily rural districts have been indicated with vertical lines.

The island of Zanzibar is a landmass of approximately 2460 km^2^ that is situated 35 kilometres (km) off the coast of east–central Africa and consists of six administrative districts. Each district is further divided into various shehias, which are administrative wards containing one or more villages. Of the six districts, two (the ‘Urban’ and ‘West’ districts) are primarily urban areas that are characterised by higher population densities and more developed infrastructure. The other four districts (the ‘North A’, ‘North B’, ‘Central’, and ‘South’ districts) are primarily rural areas that are characterised by lower population densities and less developed infrastructure [37] (Figure 1).

### 2.3. Development of the Standard Operating Procedure 

To provide technical assistance to the animal health professionals tasked with using the LFDs for diagnostic screening in this investigation, a best practice SOP termed the ‘Rapid In-field Diagnosis and Epidemiology of Rabies’ (RAIDER) toolkit was developed by the Global Alliance for Rabies Control (GARC) (File S1). The RAIDER toolkit consists of a series of established ‘best practice’ protocols that guide end-users in the steps required to collect a sample in the field, subject it to diagnostic screening with a lateral flow device, record the result and sample information (including the exact GPS coordinates) using a mobile phone application, and finally, to package and send the sample to the nearest diagnostic laboratory for confirmatory testing with a WOAH-recommended assay. The RAIDER toolkit promotes utilising LFDs in a manner that allows LFDs to confirm the presence of rabies with reasonable confidence (enabling timely disease intervention efforts such as dog vaccinations), but does not allow a negative result to be considered as conclusive until confirmed in the laboratory (see below). The required actions of the end user, according to the outcome of the LFD in-field diagnostic screening, are outlined below (the instructions provided with the RAIDER toolkit can be found in File S1).

1. Samples collected from animals involved in human exposures are expedited for diagnostic confirmation using a WOAH-recommended diagnostic assay, regardless of the diagnostic screening result. However, if the LFD result is rabies-positive, disease intervention efforts, such as outbreak responses and dog vaccinations, can be implemented while diagnostic confirmation is pending. Again, in all human exposure cases, diagnostic confirmation is prioritised to ensure the shortest turnaround time that can practically be achieved.
2. Samples collected from animals not involved in human exposures that are negative or inconclusive during the in-field diagnostic screening process are expedited for diagnostic confirmation using a WOAH-recommended diagnostic assay before any further action is taken (if necessary).
3. Samples collected from animals not involved in human exposures that test positive during the in-field screening process are treated as rabies-positive so that disease intervention efforts can be implemented in a timely manner. Such LFD rabies-positive samples are still sent for diagnostic confirmation using a WOAH-recommended diagnostic assay.

### 2.4. Implementation of the ‘Rapid In-Field Diagnosis and Epidemiology of Rabies’ Toolkit in Zanzibar

Prior to the routine implementation of the RAIDER toolkit, 20 District Veterinary Officers (DVOs) were provided with hands-on training in the different protocols of the RAIDER toolkit described below. Thereafter, the DVOs were responsible for the implementation of the active surveillance program by collecting samples from suspect animals, roadkill, and animals found dead in their respective districts before subjecting them to diagnostic screening and sending them for subsequent diagnostic confirmation at the ZCVL. In aid of this, programmatic support was provided by GARC in the form of the ADTEC LFDs and an annual contribution towards the fuel for the surveillance officers (an annual contribution that had been ongoing since 2016 when the passive surveillance system was established). All other costs associated with surveillance activities (i.e., staff costs, vehicle upkeep, etc.) were borne by the Government of Zanzibar, as has been the case since 2016.

#### 2.4.1. Collection of Animal Brain Samples

Brain samples were collected from each animal in the field using a rigid straw that was inserted through the foramen magnum, as described elsewhere [17,38]. While this has been the primary method of brain sample collection in Zanzibar since the passive surveillance started in 2016 [8] (with multiple refresher training sessions being undertaken between 2016 and 2022), refresher training was given to all the DVOs, as outlined here. Briefly, composite samples were collected by inserting the straw diagonally through the occipital foramen in the direction of both eyes, as well as in a straight line to collect brain samples from in-between the eyes. In so doing, samples were collected from the base of the Ammon’s horn, cerebellum, cortex, and medulla oblongata for each animal. After removing the composite sample, it was deposited into an appropriately labelled sample collection tube and homogenised thoroughly before being subjected to in-field diagnostic screening.

#### 2.4.2. In-Field Diagnostic Screening Using a Lateral Flow Device

In-field diagnostic screening was undertaken using the ADTEC LFD, as per the manufacturer’s instructions. Approximately one gram of mixed brain sample was transferred to a 1.5 mL microtube containing 400 µL of the assay buffer. Once transferred to the assay buffer, the sample was homogenised using a microtube pestle. The LFD was removed from the foil pouch, labelled with a field sample number, and placed on a flat, dry surface. Using the supplied dropper, approximately 100 µL of the homogenate was added to the sample hole of the LFD. After 15−20 min, the appearance of coloured line(s) were noted.

The diagnostic screening results were interpreted as follows: positive (presence of two bands, one under ‘Control’ in the result window and the other under ‘Test’ in the result window), negative (presence of one band under ‘Control’ in the result window), and inconclusive (absence of a band under ‘Control’ in the result window, regardless of whether a band appeared under ‘Test’ or not).

#### 2.4.3. Recording the Diagnostic Screening Results

After the diagnostic screening was concluded, a photograph was taken of the used ADTEC LFD before the results were captured using the Rabies Case Surveillance (RCS) tool on the GARC mobile phone application [4,8,39]. Briefly, the RCS tool was used to capture the (i) GPS coordinates, (ii) date, (iii) animal species (Dog/Cat/Livestock/Wildlife/Bat/Unknown species), (iv) field sample number, and (v) diagnostic screening result for each sample. Once the data had been captured using the RCS tool, the information was sent to the Rabies Epidemiological Bulletin (REB), where it was automatically analysed and visualised on the password-protected website that could be accessed by stakeholders from the Department of Livestock Development at any point in time [4,8,39].

### 2.5. Diagnostic Confirmation

All screened samples, as well as their corresponding ADTEC LFDs, were sent to the ZCVL for diagnostic confirmation, regardless of the diagnostic screening results. As per the RAIDER toolkit, the urgency with which the samples were sent for diagnostic confirmation depended on the outcome of the LFD, as well as the conditions which led to the animal being subjected to diagnostic screening (e.g., roadkill, euthanised in response to human exposures, etc.). Once at the ZCVL, the samples were blindly re-tested using the WOAH-recommended DRIT assay, as described elsewhere [40].

### 2.6. Resolving Diagnostic Incongruities

Samples that produced diagnostic incongruities between the DRIT assay and ADTEC LFD were re-tested with both assays (DRIT and LFD) at the ZCVL to confirm the relevant diagnostic results. 

### 2.7. Data Analysis

To determine whether the increase in sample submissions observed during the study period was statistically significant, a one-way analysis of variation analysis (ANOVA) was performed to determine the differences in the mean sample submissions per month prior to the onset of the study (July 2016–February 2022) and within the study period (March 2022–December 2023) [41]. Furthermore, to determine whether the increase in sample distribution observed during the study period was statistically significant, a one-way ANOVA was performed to determine the differences in the mean number of shehias from where samples had originated prior to the onset of the study (July 2016–February 2022) and within the study period (March 2022–December 2023) [41]. The diagnostic sensitivity, specificity, and respective confidence intervals of the ADTEC LFDs and DRIT assay were determined using an exact binomial distribution (MedCalc 12.2.1.0, Ostend, Belgium).

## 3. Results

### 3.1. Sample Submission and Distribution

**Figure 2:**
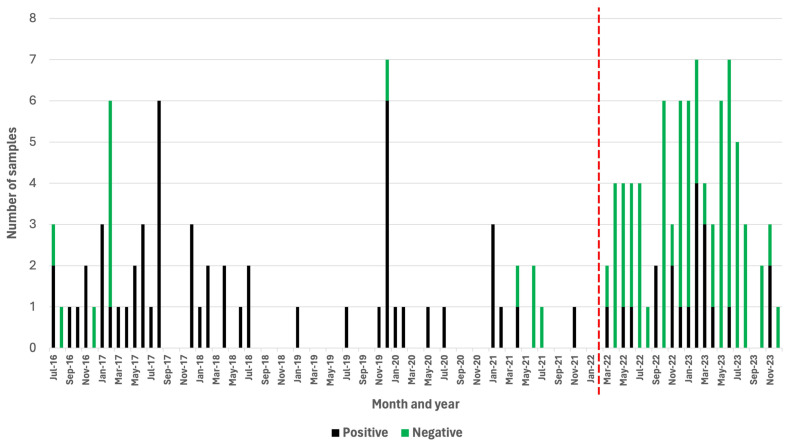
Positive and negative samples subjected to rabies diagnosis before (July 2016–February 2022) and after (March 2022–December 2023) the RAIDER toolkit had been implemented. Months where no vertical bars are present indicate a month where no samples were submitted for rabies diagnosis. The red vertical line denotes the start of the study period.

Over the course of a 68-month period (July 2016–February 2022), a passive rabies surveillance program that relied on the DRIT assay alone was implemented in Zanzibar. During this time, the ZCVL received and diagnosed a total of 67 samples originating from dogs (n = 29), goats (n = 14), cats (n = 10), wildlife species (n = 10), cows (n = 2), donkeys (n = 1), and rodents (n = 1) (Table S1). Of those, 54 (81%) were rabies-positive (Table S1). During this period, the laboratory received an average of one sample per month, while experiencing a total of 34 months (50%) where no samples were submitted for rabies diagnosis (Figure 2). During the first 22 months of implementing the active surveillance program relying on the RAIDER toolkit (March 2022–December 2023), 83 samples were screened in-field and subsequently subjected to laboratory confirmation (Table S2). These brain samples originated from dogs (n = 50), cats (n = 22), wildlife species (n = 5), pigs (n = 2), goats (n = 1), cows (n = 1), bats (n = 1), and rodents (n = 1) (Table S2). Of these, 20 (24%) were rabies-positive and 63 (76%) were rabies-negative (Table 1 and Table S2).

During the 22-month study period, there was only one month (5%) where no samples were submitted for rabies diagnosis (Figure 2). Based on these findings, the number of samples collected during the first 22 months of the study increased to an average of four samples per month (Figure 2), which constituted a statistically significant four-fold increase in the monthly number of samples submitted for rabies diagnosis (*p* = 0.00001).

While the monthly number of samples submitted for rabies diagnosis immediately started to increase with the implementation of the RAIDER toolkit, the number of negative samples increased, as one would expect for an active surveillance approach. Significantly, however, the number of rabies-positive samples collected per month during the 22-month period of active surveillance also increased (0.91 cases per month) in comparison with the number of rabies-positive samples collected during the 68-month period of passive surveillance (0.79 cases per month).

**Figure 3:**
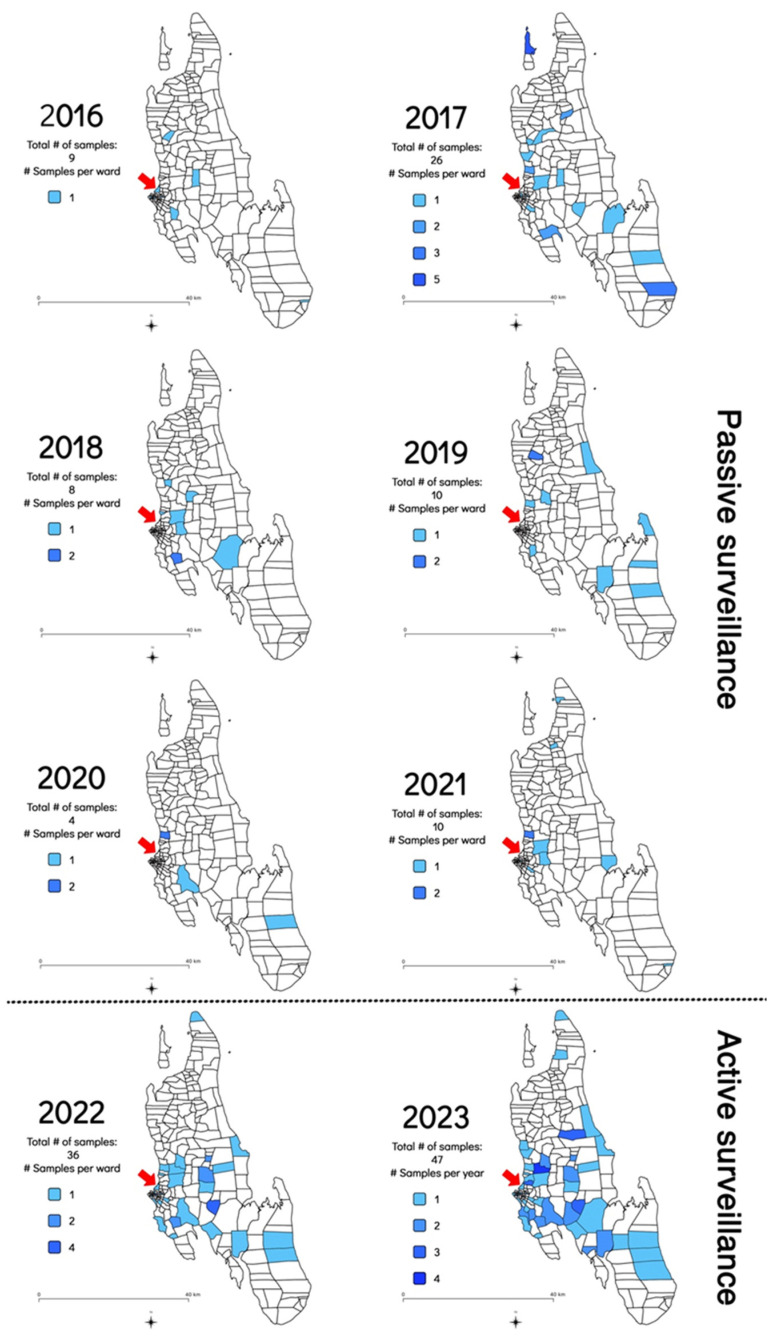
Map of Zanzibar indicating the shehias where samples were collected between July 2016 and December 2023 as shaded areas as well as the shehias where the Zanzibar Central Veterinary Laboratory is located with a red arrow. The 2016 map only included data collected from July to December 2016 as no rabies diagnostic capabilities existed before that date [8]. The 2022 map only included data generated during the implementation of the RAIDER toolkit (March–December 2022) as no samples had been collected as part of the passive surveillance work in January and February of that year.

In addition to the increase in the frequency of sample submission and rabies cases diagnosed, the geographical distribution of the samples submitted for rabies diagnosis was also investigated to determine whether the implementation of the RAIDER toolkit resulted in samples being collected from more shehias across the island. Between July 2016 and February 2022, samples originated from an average of nine shehias per year (Min: 3 in 2020; Max: 17 in 2017) (Figure 3 and Table S1). In contrast, between March 2022 and December 2023, samples originated from an average of 31 shehias per year (Min: 29 in 2022; Max: 32 in 2023) (Figure 3 and Table S2). This constituted a statistically significant three-fold increase in the number of shehias where samples were collected and diagnosed (*p* = 0.001235).

### 3.2. Targeted Dog Vaccinations in Response to Diagnostic Screening Results

**Figure 4:**
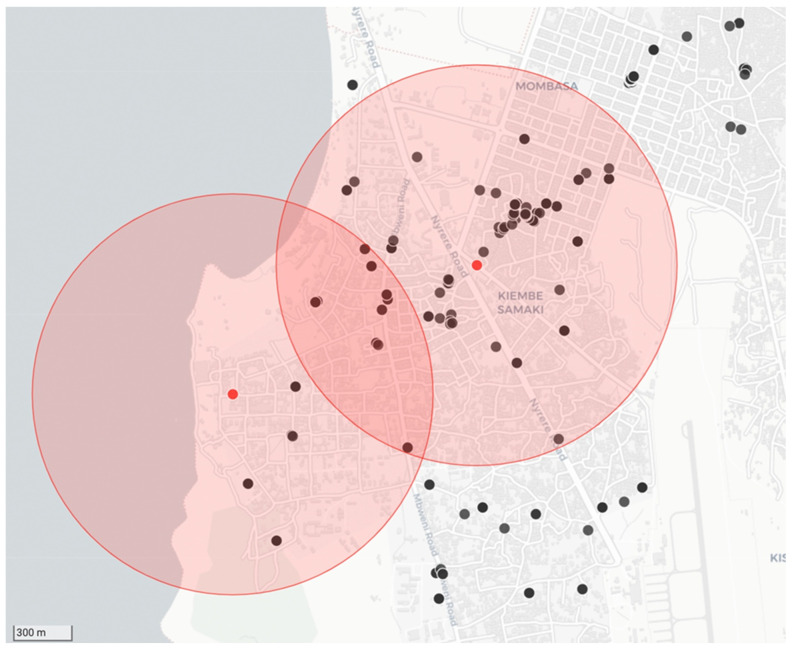
Example of the data collected during a strategic dog vaccination campaign that was initiated in response to positive diagnostic screening results. The location of the two samples that tested positive for rabies are represented by red dots with a 1 km catchment area shown around each of them as shaded red circles. The black dots show the locations where dogs were vaccinated in response to the cases.

As per the recommendations provided in the RAIDER toolkit, samples that tested positive during the diagnostic screening process were treated as such while awaiting diagnostic confirmation. Using the diagnostic screening results and exact GPS coordinates captured by the RCS tool on the mobile phone application, vaccination campaign coordinators could easily identify the locations of samples that tested positive for rabies during the diagnostic screening process (n = 20) with the use of the interactive maps on the REB (Figure 4). With the aid of this real-time data, timely targeted dog vaccinations in and around the communities where the samples tested positive were routinely undertaken—with the dog vaccination data being recorded on the same mobile phone application (Figure 4).

### 3.3. Statistical Analysis of the Diagnostic Efficacy of the ADTEC LFD

The number of true-positive (n = 20) and -negative (n = 63) samples (determined by the DRIT assay) were used to calculate the diagnostic efficacy of the ADTEC LFD (Table 2). Based on the confirmatory results provided by the DRIT assay, the ADTEC LFD produced one false-negative result (DRIT-positive but LFD-negative; n = 1/83). As a result, the ADTEC LFD had a diagnostic sensitivity of 95% and specificity of 100% when applied to the cohort of samples in this study (Table 2).

## 4. Discussion

The elimination of canine-mediated human rabies has become an important objective for global bodies concerned with One Health and Neglected Tropical Diseases, among others [42]. The ultimate key to evaluating the success of rabies elimination efforts lies in robust and accurate disease surveillance. Without this, there can be no true measurement of impact and no reliable data to guide or improve elimination programmes. It is, therefore, critical that rabies-endemic countries striving for rabies elimination must implement robust rabies surveillance systems that facilitate the collection and timely diagnosis of samples from across the country [4,5,6,10]. These efforts are not only vital for routine and targeted disease control efforts, but also for the eventual self-declaration of freedom from canine-mediated rabies [43]. However, in striving to improve surveillance programmes, countries have to factor in that robust rabies surveillance is cumbersome, expensive, and reliant on well-equipped professional laboratories and infrastructure for the effective transport of hazardous and labile samples or carcasses. In this regard, it could be argued that LFDs might potentially be quite useful considering their ease-of-use, cost effectiveness, practicality, and appeal in terms of routine implementation. Indeed, it is, therefore, hardly surprising that the reported use of lateral flow devices (LFDs) is rapidly increasing [10,12,18,19,20]. While the diagnostic capabilities of different rabies LFDs have undergone extensive scientific scrutiny—and their shortcomings have been well documented [12,18,20,21]—none of the studies published to date have determined the impact that LFDs could make towards improving rabies surveillance networks by providing point-of-care diagnostic screening. As such, this study endeavoured to demonstrate the benefits that LFDs could provide to enhance rabies surveillance networks if their routine use is managed through strict guidelines and recommendations. In so doing, this would provide much-needed guidance to animal health professionals who wish to integrate (or have already integrated) LFDs into their rabies surveillance activities. More specifically, we report here on the development, implementation, and benefits of the RAIDER toolkit, which was used to ensure the judicious use of LFDs as a diagnostic screening tool in Zanzibar over the course of a 22-month period. 

In this study, the ADTEC LFD correctly diagnosed 82 samples (19 rabies-positive and 63 rabies-negative) and produced a single false-negative result. Due to the single false negative result, the diagnostic sensitivity and specificity of the ADTEC LFD were 95% and 100%, respectively (Table 2). This reported diagnostic sensitivity was similar to that observed when the ADTEC LFD was implemented in the Philippines (94% to 100%) [19,32,33]. Furthermore, our results suggested that the ADTEC LFD was comparable to another commercially available LFD (Anigen Rapid Rabies Ag Test Kit, Bionote, Republic of Korea) that is widely used and also has a diagnostic sensitivity of approximately 95% based on published findings [19,23,25,26,31,44].

As discussed, the ADTEC LFD results generated in this study were not in total agreement with those produced by the WOAH-recommended DRIT assay. While the occurrence of a false-negative result was not ideal from a public health perspective, it should be noted that the use of the RAIDER toolkit not only ensured that all the samples were eventually tested with a WOAH-recommended diagnostic assay for official disease reporting purposes, but that samples that were LFD-negative (such as the one discussed here) were prioritised for confirmatory testing at the ZCVL. As a result, there was no significant delay between the diagnostic screening outcome and obtaining the correct diagnostic result through confirmatory testing.

It had been suggested elsewhere that a slight sacrifice in the sensitivity of diagnostic tests should be considered acceptable if their use makes a positive impact on the robustness of the overall surveillance program [10]. While some previous studies have alluded to the fact that LFDs could fulfil such a role by encouraging the collection and testing of samples [23,26], none investigated this impact in earnest. In fact, only one study in Namibia attempted to determine the benefits that LFDs provided to the reach of surveillance networks, with the authors anticipating an increase in the level of surveillance using the LFD before diagnostic confirmation at the central veterinary laboratory. That study was limited in the fact that not all the samples subjected to diagnostic screening with the LFDs were sent for subsequent diagnostic confirmation, with results going unreported as a result thereof [29].

In our study, we found that the integration of the LFDs, coupled with the guidance provided through the RADIER toolkit, had a positive and noteworthy impact on the robustness of the surveillance network in Zanzibar. More specifically, the LFDs resulted in a four-fold increase in the number of samples subjected to rabies diagnosis per month, a three-fold increase in the number of shehias where samples were collected per year, and a marked (and sustained) increase in cases diagnosed. In addition, the use of the LFDs positively impacted the government’s ability to respond to outbreaks by initiating precise targeted dog vaccination campaigns without delay (Figure 4). While we can only speculate as to why the LFDs were so well received by the DVOs in this investigation (resulting in sustained implementation of the LFDs during the entire project period), some informal feedback suggested that the ability to provide the community with more information on site (point-of-care) was a noteworthy advantage that encouraged the continued use of the LFDs during, as well as after, the study period. It should be stated that, to avoid any obvious sampling biases that could influence the results reported here, the implementation of the RAIDER toolkit benefited from no additional external financial support towards the operational costs of the surveillance activities compared to the years prior to this investigation.

## 5. Conclusions

In conclusion, the findings presented here suggest that LFDs could play a noteworthy role in improving the robustness of surveillance systems by enabling more samples to be tested from broader geographical areas, most notably, those areas distant from the rabies diagnostic laboratory. Their implementation would, however, need to be carefully controlled through standardised protocols (such as the RAIDER toolkit) that align with international best practices. In so doing, LFDs could be integrated into surveillance systems where they are used to rapidly detect and report on rabies cases in a field setting for timely outbreak responses, while diagnostic confirmation continues to play a role in terms of both confirming results and official data-reporting purposes. This approach would enable national authorities to start, or continue, using LFDs in an acceptable manner—improving the collection of empirical disease burden data and gaining support for disease elimination strategies that could meaningfully progress rabies-endemic countries in their aspirations to become free of canine-mediated human rabies.

## Figures and Tables

**Table 1:** Active surveillance with the RAIDER toolkit: Overview of the reasons why diagnostic screening was implemented as well as the final diagnostic outcome.

Reason for Diagnostic Screening	Rabies Positive (%)	Rabies Negative (%)
Animal was showing signs of rabies and was humanely euthanised	17 (20%)	38 (46%)
Animal was showing signs of rabies and was killed by community members	3 (4%)	4 (5%)
Roadkill	0	16 (19%)
Animal found dead	0	5 (6%)

**Table 2:** Diagnostic sensitivity and specificity of the ADTEC LFD applied to a cohort of samples collected in Zanzibar.

	True Positive	False Positive	True Negative	False Negative	Diagnostic Sensitivity (95% CI)	Diagnostic Specificity (95% CI)
DRIT	20	0	63	0	100% (83.16–100%)	100% (94.31–100%)
ADTEC LFD	19	0	63	1	95% (75.13–99.87%)	100% (94.31–100%)

CI: confidence interval.

## Data Availability

Surveillance data on rabies cases that support the findings of this study are available in the supplementary data. The original contributions presented (through figures and tables) in the study are included in the article/supplementary material and further inquiries can be directed to the corresponding author.

## Supplementary Materials

The following supporting information can be downloaded at: https://www.mdpi.com/article/10.3390/microorganisms12071314/s1, Table S1: Neuronal tissue sample cohort from Zanzibar depicting the diagnostic confirmation results using the DRIT assay at the Zanzibar Central Veterinary Laboratory; Table S2: Neuronal tissue sample cohort from Zanzibar depicting the initial in-field diagnostic screening results and their diagnostic confirmation using the DRIT assay at the Zanzibar Central Veterinary Laboratory; Figure S1: The ‘Rapid In-field Diagnosis and Epidemiology of Rabies’ toolkit.
