# Supplementary material for: Investigating the Impact That Diagnostic Screening with Lateral Flow Devices Had on the Rabies Surveillance Program in Zanzibar, Tanzania
Source: Microorganisms. 2024 Jun 27;12(7):1314. doi: 10.3390/microorganisms12071314 (PMC11279036; doi:10.3390/microorganisms12071314)
Supplement: Supplementary file 1 [file microorganisms-12-01314-s001.zip › File S1.pdf]

Rapid In-field Diagnosis and Epidemiology of Rabies (RAIDER) toolkit.

LFDs: ADTEC's "Rabies Ag test"

Table of Contents

ABBREVIATION LIST 2

OVERVIEW: RAIDER TOOLKIT 3

SITUATIONAL IMPLEMENTATION OF THE RAIDER TOOLKIT 4

SAMPLE COLLECTED FROM A DEAD ANIMAL (MAMMAL) FOUND IN THE COMMUNITY 4

SAMPLE COLLECTED FROM A BITING ANIMAL (MAMMAL) THAT HAS DIED 5

OVERVIEW OF RAIDER TOOLKIT PROTOCOLS 6

STEP 1 - BRAIN REMOVAL (PROTOCOL) 7

OVERVIEW: 7

MATERIALS: 7

METHODOLOGY: 7

NOTES: 10

STEP 2 - RABIES DIAGNOSIS WITH ADTEC'S "RABIES AG TEST" (PROTOCOL) 12

OVERVIEW: 12

MATERIALS: 12

METHODOLOGY: 12

NOTES: 15

STEP 3 - REPORTING THE RESULT USING THE RCS TOOL ON THE GARC APP (PROTOCOL) 16

OVERVIEW: 16

MATERIALS: 16

METHODOLOGY: 16
SENDING THE CASE INVESTIGATION DATA TO THE WEBSITE 35
STEP 4 - SAMPLE STORAGE AND SHIPMENT TO LABORATORY (PROTOCOL) 38
OVERVIEW: 38
MATERIALS: 38
METHODOLOGY: 39

Abbreviation list

DHIS2	District Health Information System 2
GARC	Global Alliance for Rabies Control
GPS	global positioning system
LFD	lateral flow device
WOAH	World Organisation for Animal Health
PBS	phosphate buffered saline
RAIDER	Rapid, In-field Diagnosis and Epidemiology of Rabies
REB	Rabies Epidemiological Bulletin
RCS	Rabies Case Surveillance

Overview: RAIDER toolkit

Detecting a mammalian carcass initiates the RAIDER toolkit protocol.

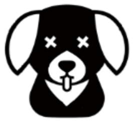

Protocol 1

A brain specimen is collected from the carcass using a safe, non-invasive technique.

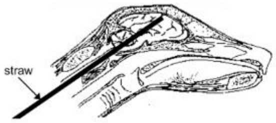

Protocol 2

The sample is tested with ADTEC's "Rabies Ag test" LFD

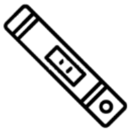

Protocol 3

The diagnostic result is recorded on the GARC App for further analysis using the Rabies Epidemiological Bulletin (REB).

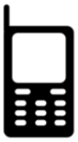

Protocol 4

The sample is packaged and sent to the nearest laboratory.

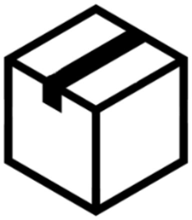

Targeted rabies vaccination takes place where rabies was detected.

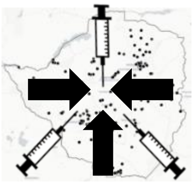

Protocol 5

Once at the laboratory, the sample is subjected to diagnostic confirmation and the data on the REB is updated if required.

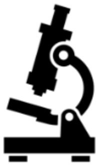

Situational implementation of the RAIDER Toolkit

Sample collected from a dead animal (mammal) found in the community

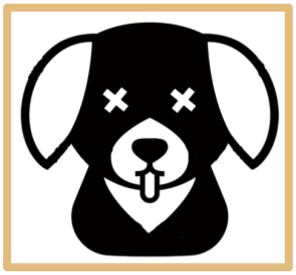

- Collect a brain sample from the carcass and test it using ADTEC's "Rabies Ag test" (LFD).
- **If the result is positive for rabies**: record the result on the RCS tool on the GARC App and send the sample to the laboratory for diagnostic confirmation **once an opportunity arises**.
- **If the result is negative or inconclusive for rabies**: record the result on the RCS tool on the GARC App and send the sample to the laboratory for diagnostic confirmation **as soon as possible**.
- The carcasses should be **burnt, composted, or buried** if rabies is suspected.
- People should be advised not to butcher, handle or consume meat from a suspected rabid animal.

Sample collected from a biting animal (mammal) that has died

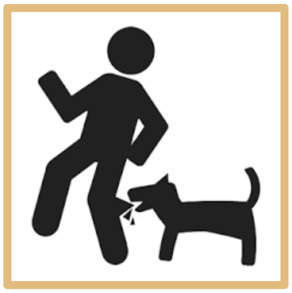

- Collect a brain sample from the carcass and test it using ADTEC's "Rabies Ag test" (LFD).
- **If the result is positive for rabies**: record the result on the RCS tool on the GARC App and send the sample to the laboratory for diagnostic confirmation **as soon as possible**.
- **If the result is negative or inconclusive for rabies**: record the result on the RCS tool on the GARC App and send the sample to the laboratory for diagnostic confirmation **as soon as possible**.
- The carcasses should be **burnt, composted, or buried** if rabies is suspected.
- People should be advised not to butcher, handle or consume meat from a suspected rabid animal.

Overview of RAIDER toolkit protocols

The protocols listed below outline the RAIDER toolkit and should be followed from Step 1 to Step 4 without interruption. Step 5 will be completed after a period of time, as this requires shipment and laboratory diagnosis. As such, while the field methodology (Step 1 – 4) relies on four distinct protocols, they are part of one process.

Step 1: Brain removal using a straw

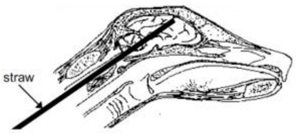

Step 2: Rabies diagnosis with ADTEC's "Rabies Ag test" (LFD)

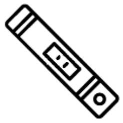

Step 3: Reporting the results using the RCS tool on the GARC App

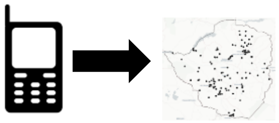

Step 4: Sample storage and shipment to the nearest laboratory

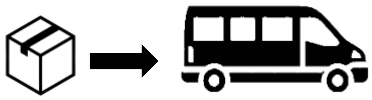

Step 5: Updating the sample information after diagnostic confirmation

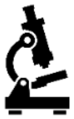

Step 1 – Brain removal (protocol)

Overview:

Collecting a brain sample by opening the skull is hazardous when conducted under field conditions. In such circumstances, a brain sample can be collected without opening the skull, by accessing the brain material through the foramen magnum.

Materials:

- Straight drinking straws (≥ 5mm in diameter) or Pasteur pipettes with the tip cut diagonally to create a pointed end (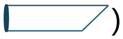) before being used in the field.
- Disposable scalpels
- Rigid container for used scalpels (e.g., "Sharps" container)
- Nitrile/Latex gloves
- Personal protective equipment (knee-length disposable gown, protective eyewear)
- A bottle of clean water (5 litres; preferably with a tap) and liquid soap
- Disinfectant solution (bleach solution)
- Spray bottle
- Measuring cylinder
- Sample transport tube (e.g. 50ml Falcon tube)
- Waste packet
- Marker/pen
- Plastic sheet (work surface)

Methodology:

Preparation

1. Put on latex/nitrile gloves and personal protective equipment.
2. Lay down the plastic sheet on a flat piece of ground as the "work surface".
3. Make up fresh disinfectant in spray- and instrument-soaking bottles (10% bleach solution) using the water and measuring cylinder
4. Lay out all of the equipment and bottles on the plastic sheet
5. Open a new sample transport tube. Label the tube with the date, location and sample number.
6. Place the carcass on the plastic sheet

Access to the foramen magnum

7. Bend the head to expose the occipital region (*Bend the head to expose the back of the neck*).

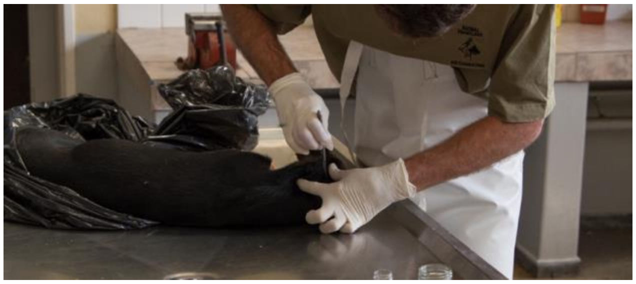

8. Cut the skin and neck muscles over the joint between the occipital bone and the atlas vertebra using a disposable scalpel (*cut from ear to ear following the line of the skull*). Be careful to avoid injury to yourself.

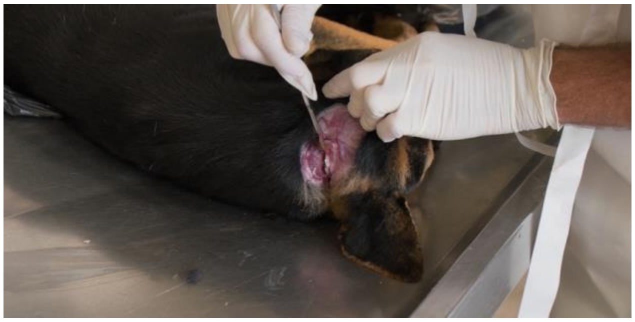

9. Open the atlanto-occipital joint by cutting the dorsal membrane and the meninges to access the foramen magnum (*cut between the last vertebrae and skull to expose the base of the skull and the spinal cord*).

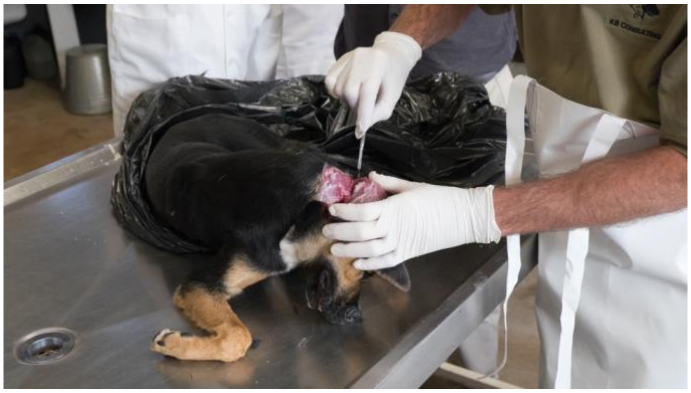

10. Discard the scalpel into the disposal container (**Do not put the sheath back on the scalpel blade)**.

Collection of brain sample

11. Enter the straw through the foramen magnum (*hole where the spinal cord enters the base of the skull*) and turn while pushing it towards **the one eye** (e.g., left eye). By turning/screwing the straw, a comprehensive sample is obtained as you move through the brain matter.

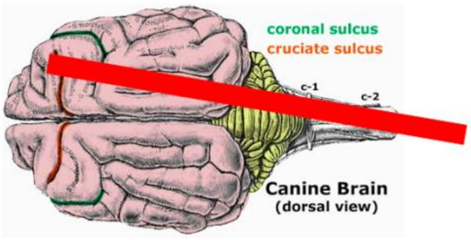

12. Close the top of the straw with a finger and gently withdraw it while continuing to turn (ensure that you maintain a seal at the top of the straw using your finger until you are ready to put the sample into the tube).
13. Keeping your finger on the top of the straw, enter the straw through the foramen magnum again and turn it while pushing it towards **the other eye** (e.g., right eye).

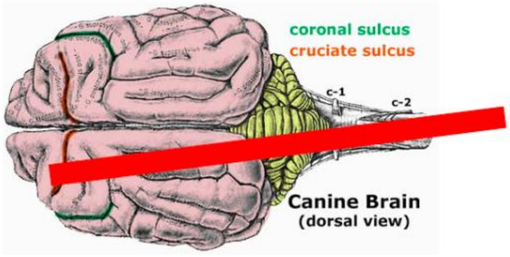

14. Gently withdraw it while continuing to turn (ensure that you maintain a seal at the top of the straw using your finger until you are ready to put the sample into the tube).
15. Keeping your finger on the top of the straw, enter the straw through the foramen magnum again and turn while pushing it towards **the area between the eyes**.

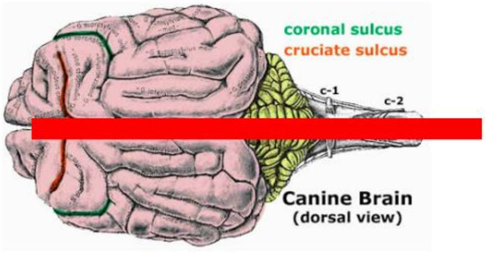

16. Gently withdraw the straw while continuing to turn (ensure that you maintain a seal at the top of the straw using your finger until you are ready to put the sample into the tube).

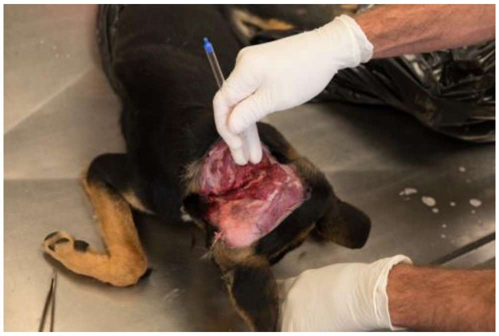

17. Hold the tip of the straw over the sample transport tube and remove your finger from the top of the straw (**The sample should slide out of the straw and fall into the sample transport tube**). If the sample does not slide out, tap the top of the straw several times with your finger until all the brain material has gone into the sample transport tube. **Note:** Step 11 – 15 can be repeated multiple times if more brain material is required.

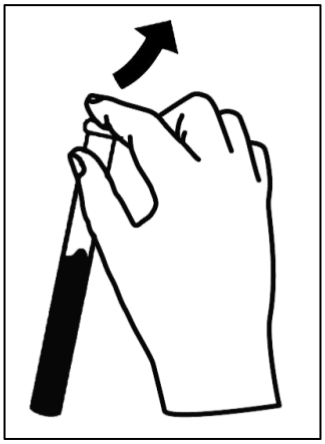

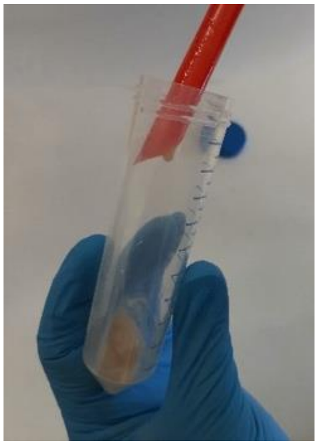

Cleaning up

18. After all brain material has slid out of the straw, the empty straw needs to be discarded into the waste packet.
19. If you need to move or dispose of the carcass, do it with your soiled gloves.
20. When handling of the carcass and sample extraction is complete, soiled gloves must be placed into the waste packet.
21. Spray the waste packet down with the disinfectant (10% bleach solution). Leave the waste packet open as it will be required for the LFD protocol that follows.
22. Wash your hands thoroughly with the clean water and soap in your kit.

Notes:

- Use one set of instruments per animal to avoid cross-contamination of specimens.

- Clean and decontaminate any contaminated instruments chemically as soon as they have been used.
- Equipment should be allowed to air dry before it is used again.
- Ensure that the sample is labelled appropriately (see relevant protocol on sample shipment).

Step 2 – Rabies diagnosis with ADTEC's "Rabies Ag test" (protocol)

Overview:

ADTEC's "Rabies Ag test" lateral flow device (LFD) is a chromatographic immunoassay that enables in-field "qualitative" detection of rabies lyssavirus antigen in brain samples taken from terrestrial mammal species.

Materials:

- Tube containing assay diluent buffer
- Wooden spatula
- Disposable droppers
- LFD test card
- Sample Masher kit* (1.5 ml tube and pestle)
- Timer
- Nitrile/Latex gloves
- Disinfectant solution (10% bleach solution) in a spray bottle
- Waste packet
- Marker/pen
- Sealable, water-tight plastic bag (e.g. Ziplock bag)

Methodology:

Preparing the sample

1. Put on a new pair of latex/nitrile gloves
2. Homogenize the sample in the sample transport tube (see the sample collection protocol for more details) using the wooden spatula to ensure that any virus particles are distributed throughout the sample.

Testing the sample

3. Collect a piece of the homogenized sample (±1gram/the size of a match head) from the sample transport tube using the wooden spatula.
4. Transfer the small piece of homogenized sample to the 1.5 mL microtube (Sample Masher kit) and add 400 µL of the Assay Buffer.

5. Homogenize completely using the pestle provided in the Sample Masher kit.
6. Remove the test device from the foil pouch. Label it clearly with the “field sample number” (provided by the program manager) and place it on a flat and dry surface.
7. Using the dropper or pipette, apply 100 µL of the homogenate supernatant to the sample hole of the test card.
8. **After 15−20 minutes, watch for the appearance of a colored line on the nitrocellulose membrane.**

Interpreting the results

Negative result:

- A distinct color band will appear in the left section of the result window to show that the test is working properly (line marked as “Control” on the result window). This is the control band.
- No line will appear in the right section of the result window (line marked as “Test” on the result window) showing that the result is negative.

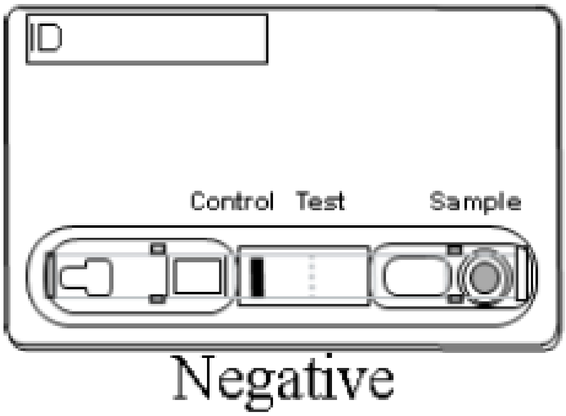

Positive result:

- A color band will appear in the left section of the result window to show that the test is working properly (line marked as “Control” on the result window). This is the control band.
- A color band will appear in the right section of the result window (line marked as “Test” on the result window) showing that the result is positive.

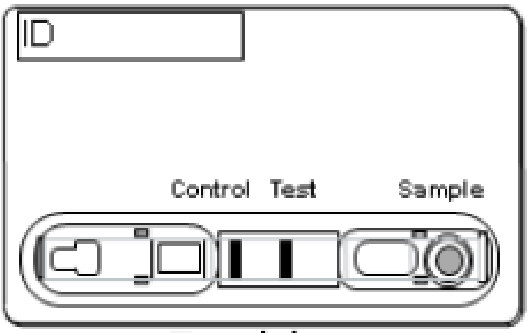

Positive

Invalid results (sample should be re-tested immediately using another LFD kit):

- No color band appears in the left section of the result window (line marked as “Control” on the result window) regardless of a band appearing in the right section of the result window.

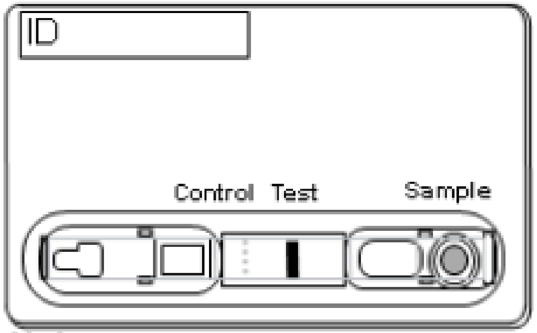

- No color bands appear in the result window.

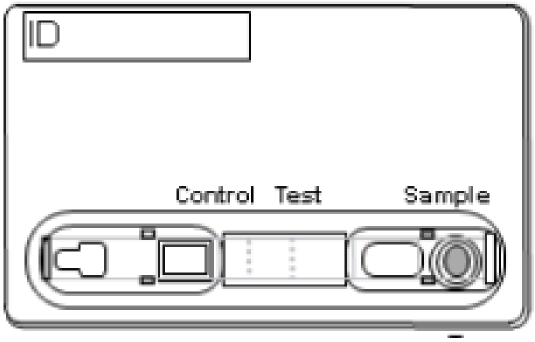

Cleaning up

9. Remove your gloves and discard them into the waste packet. Without gloves on, take a photo of the diagnostic outcome using your mobile phone camera. Be sure to include the sample number in the photo (this photo should be sent to the laboratory staff via text

messaging, instant messaging, or email for confirmation). **Important note:** Please be sure to only touch your mobile phone when not wearing gloves. DO NOT touch the LFD (rapid test kit) without gloves on.

10. Put on a new pair of latex/nitrile gloves
11. Place the used test kit in a clear sealable plastic bag and spray the outside of the bag down with disinfectant solution. Allow the disinfectant to work for 10 minutes before handling the plastic bag.
12. Place all contaminated items (droppers, wooden spatula, diluent tube, etc.) into the waste packet.
13. Place soiled gloves into the waste packet.
14. Spray the waste packet down with disinfectant.
15. Allow the disinfectant to work for 10 minutes before handling the packet.
16. Wash your hands thoroughly with the clean water and soap in your kit.

Notes:

- Each test kit can only be used once.
- The kit can be stored at room temperature or refrigerated (2 - 25°C).
- Do not freeze the kit and do not store the kit in direct sunlight.
- Do not use the kit if it has expired (see the expiration date on the pouch of the test).

Step 3 – Reporting the result using the RCS tool on the GARC App (protocol)

Overview:

The RCS tool on the GARC App allows users to report diagnostic results directly to the REB, which in-turn produces interactive spatio-temporal maps that pinpoint the locations of diagnosed rabies cases at a community-level by enabling the end-user to input the animal species, the diagnostic outcome, and the sample number for each diagnosed specimen. Other critical information such as user information, time, date, and GPS coordinates are also captured by the GARC App.

Materials:

- Mobile phone (no internet required when capturing data)
- GARC App installed in the phone

Methodology:

Opening the GARC App

1. Open your application by clicking on the App icon (DHIS2).

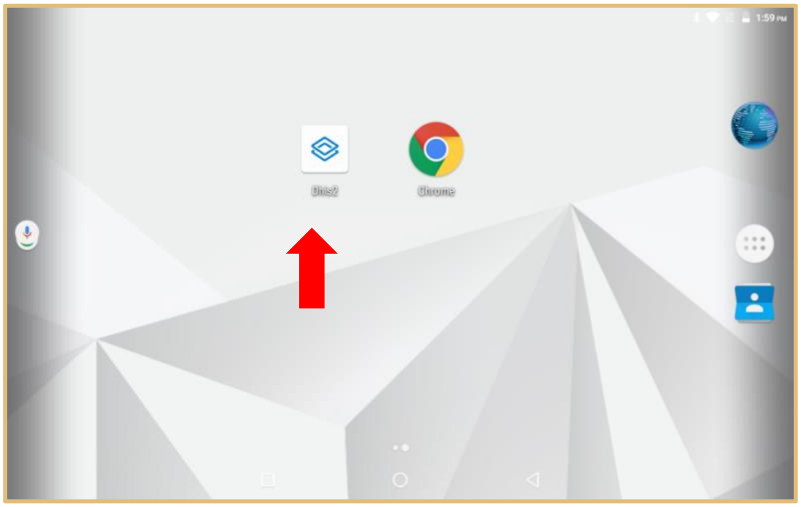
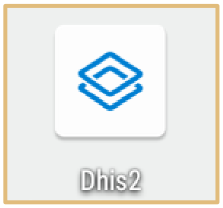

2. Enter the following address into the 'Server URL' field: https://bulletin.rabiesalliance.org
NOTE: You will only have to do this once.

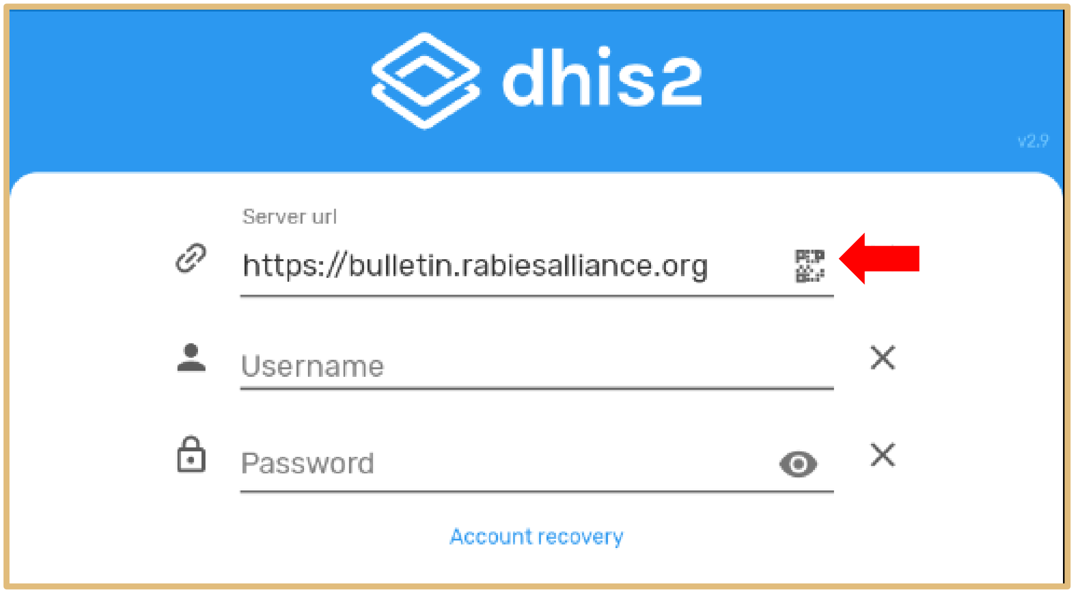

3. Enter your unique username and password (If you do not have a username or password, or encounter any problems, please contact your data manager, or project coordinator). **NOTE:** Please ensure that you type in your username and password EXACTLY as it appears in the email sent to you by the GARC administrator (including upper case letters, lower case letters, numbers and special characters).

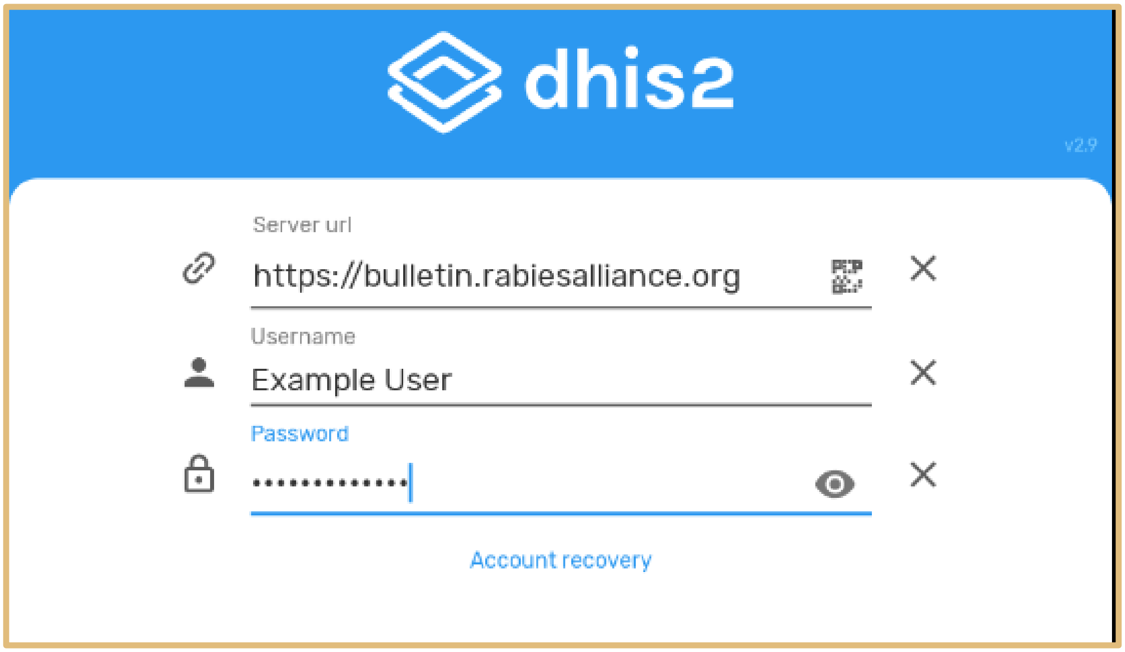

4. Click on the 'Log in' icon to access the GARC App.

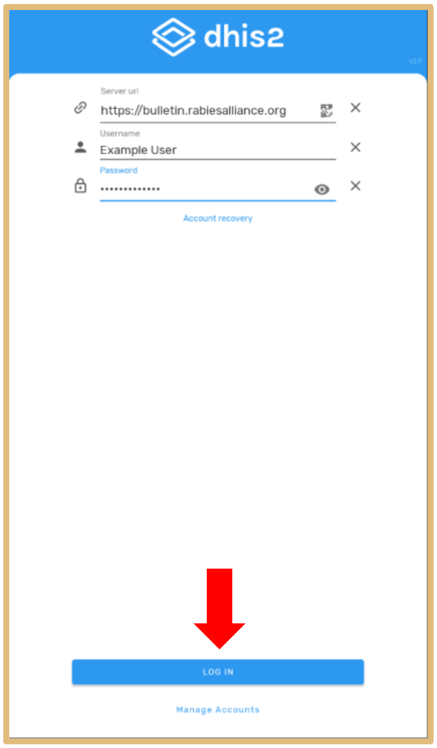

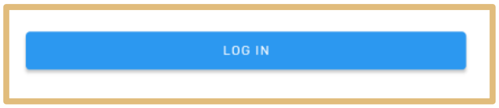

5. When you log in for the first time, you will be asked whether you want to help improve the App. You can choose any option.

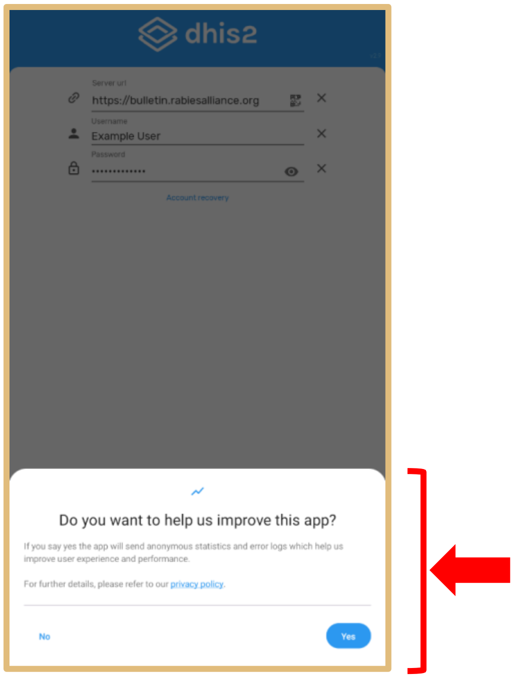

6. **The first time you access the mobile phone application you will need a stable internet connection to synchronize the mobile phone with the system. Thereafter,**

the app can be used offline. Please note that this process may take a few minutes depending on your internet connection.

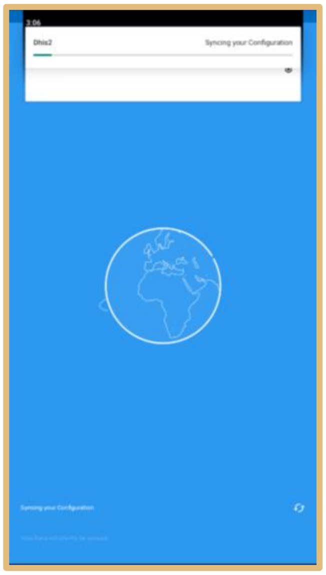

7. After synchronizing with the system, the mobile application will open and be ready for use.

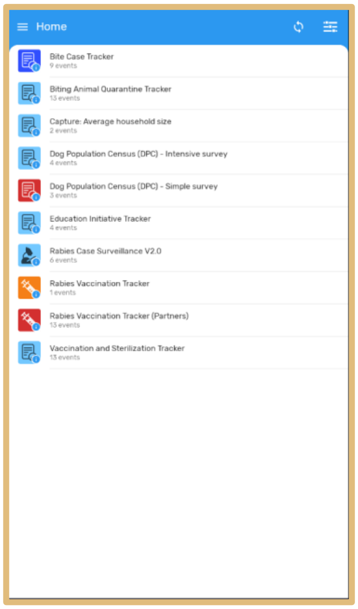

NOTE: you might not be asked to enter the username and password every time unless you 'Log out'. If you did not log out, clicking on the icon of the mobile app will automatically open the program on the device.

Capturing the rabies case surveillance data

1. Select the Rabies Case Surveillance V2.0 tool from the list. If you only have access to the Rabies Case Surveillance tool then this step can be ignored (please see point 2 below).

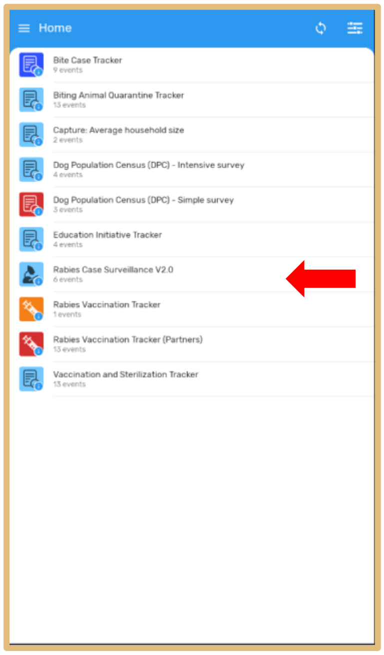

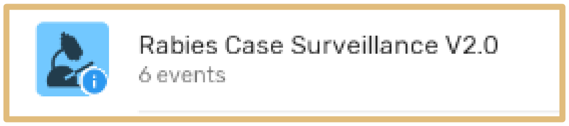

2. Click on the 'plus' sign at the bottom right of the screen to create a new entry.

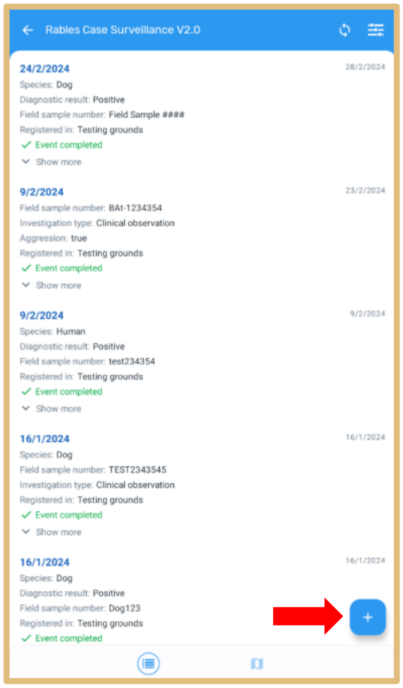

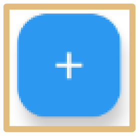

3. Ensure that the displayed date is when the rabies case was detected or diagnosed.

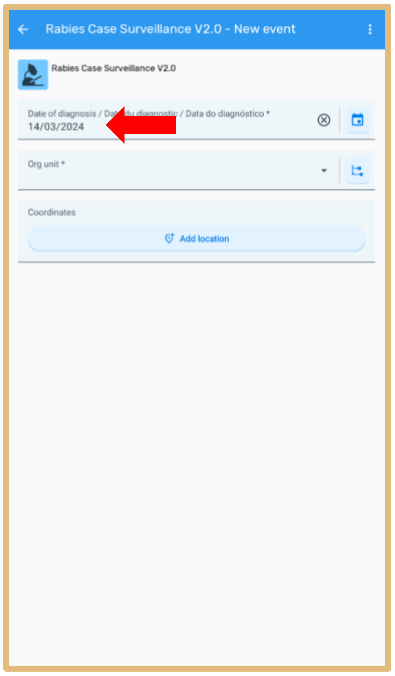

Note: If you need to change the date, you can click on the Calendar icon and select the relevant date from the calendar that opens.

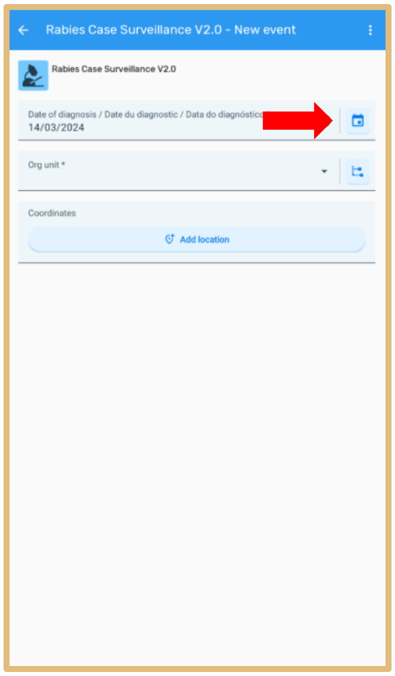

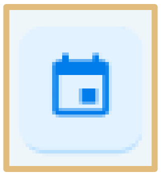

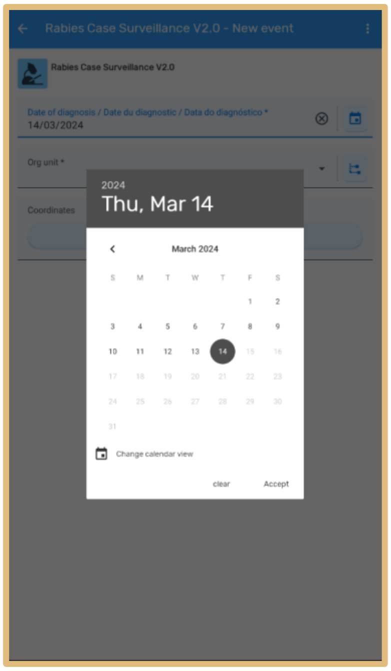

4. The ‘Org Unit’ selection tells the Rabies Epidemiological Bulletin to which administrative level (town, ward, district, etc.) the data is attributed. Click on the Org Unit and select the specific **location where the rabies case was detected** from the dropdown list. **NOTE:** always choose the lowest possible administrative level (e.g., ward or municipality) rather than a district. After selecting the relevant Org Unit, click on ‘Done’.

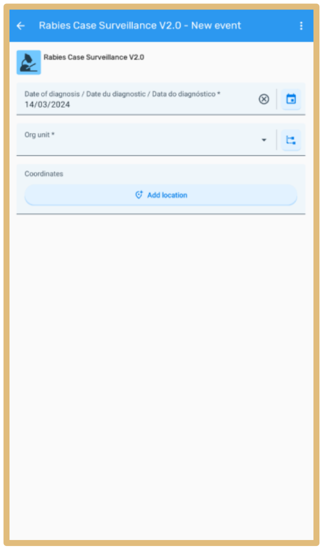

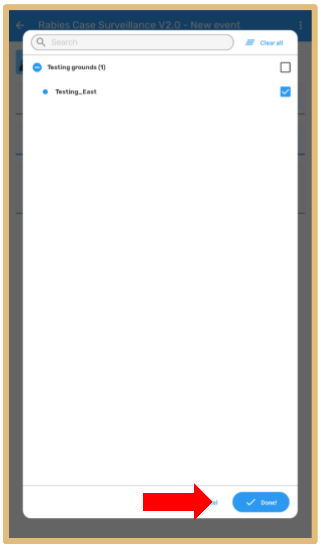

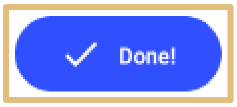

NOTE: You can also search for specific Org Units using the ‘Search’ bar at the top of the page in instances where you have access to multiple different Org Units at different administrative levels.

5. Click on the ‘Add location’ button to capture your GPS location. **NOTE: Ensure that the “Location settings” on your mobile device is turned on (this is a setting on your mobile phone and not on the app).**

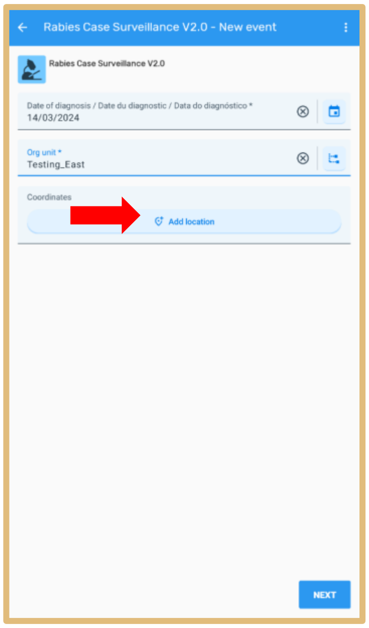

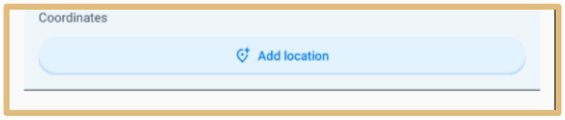

6. After the map opens, ensure that the correct location has been identified and click the ‘Done’ button. If not, you can move the pin to the correct location by touching the screen. Once the pin is accurately reflecting your location, click on the ‘Done’ button.

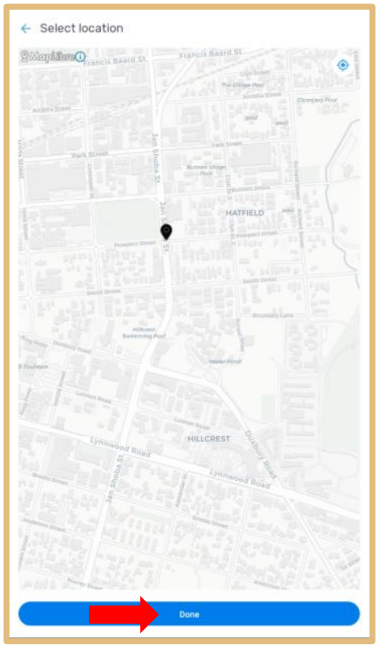

7. Confirm that the GPS coordinates have been captured and click the ‘Next’ button on the bottom right of the screen.

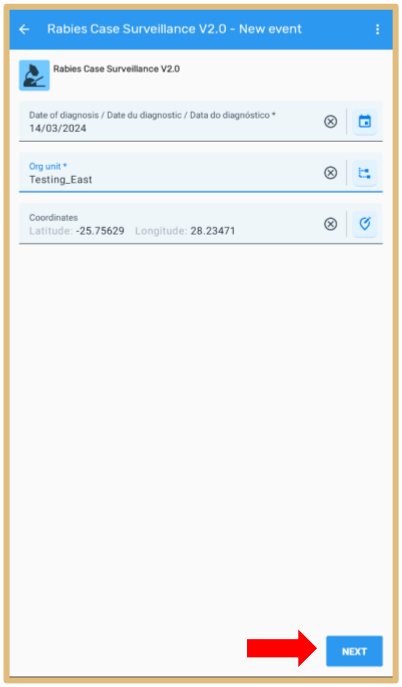

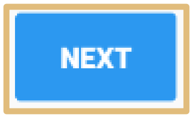

8. Select the appropriate animal species.

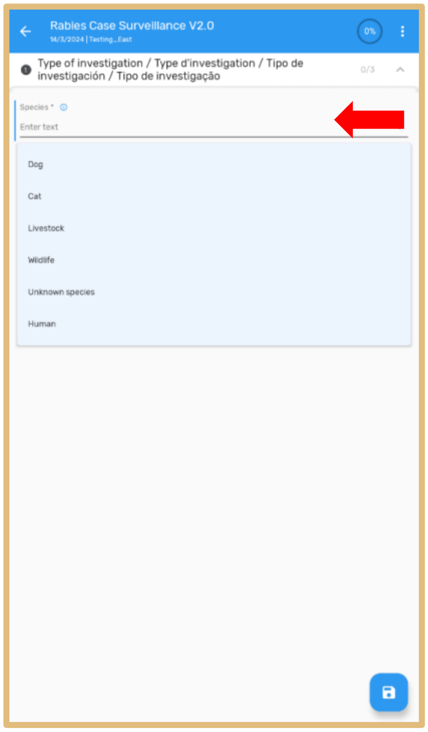

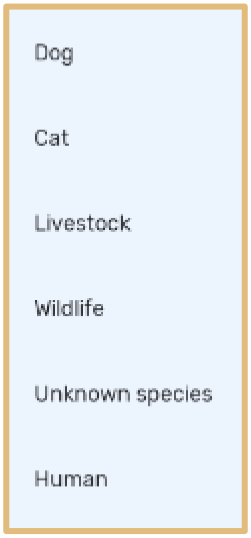

9. Enter a unique field sample number. **NOTE:** If this is a clinical diagnosis of an animal, be sure to add a record number in the "field sample number" box to later identify that animal and investigation. If a sample was taken for laboratory diagnosis, it is very important to include a field number so that the record can be found and updated at the laboratory later. **Make sure to write the same field sample number on any paper-based sample submission forms that will go to the laboratory along with the sample.**

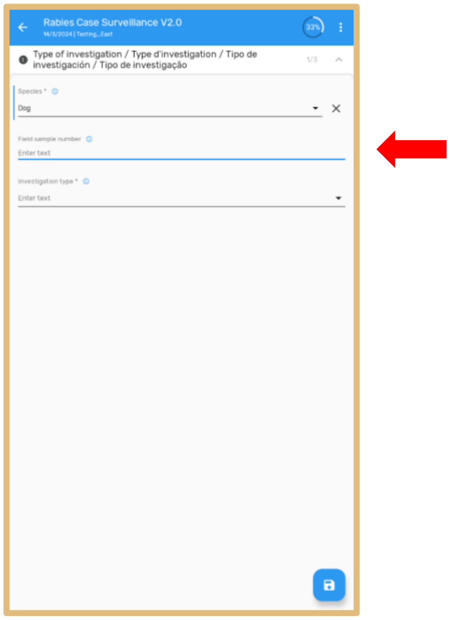

10. Select the investigation type from the drop-down menu.

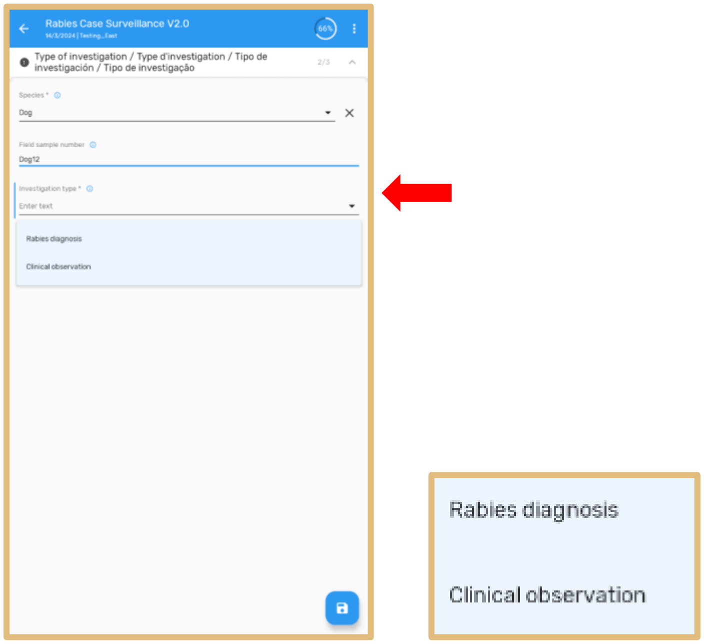

11. Different sections of the form will open depending on whether ‘Rabies diagnosis’ or ‘Clinical observation’ was selected under ‘Investigation type’. See the relevant sections below detailing each section.

    NOTE: Select ‘Rabies Diagnosis’ if you are capturing a diagnostic screening (e.g., LFD or Rapid Test Kit) or laboratory confirmation result. Select ‘Clinical observation’ if you are ONLY capturing observational data on the signs of rabies observed.

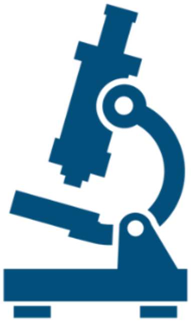	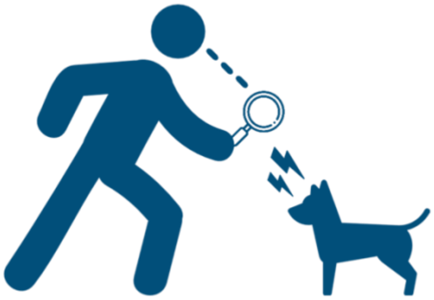
Go to: Capturing rabies diagnostic data	Go to: Capturing clinical observation data only

Capturing rabies diagnostic data

12. When selecting ‘Rabies diagnosis’ under ‘Investigation type’, two additional sections of the form will open: ‘Rabies diagnosis’ and ‘Clinical observation in animals’.

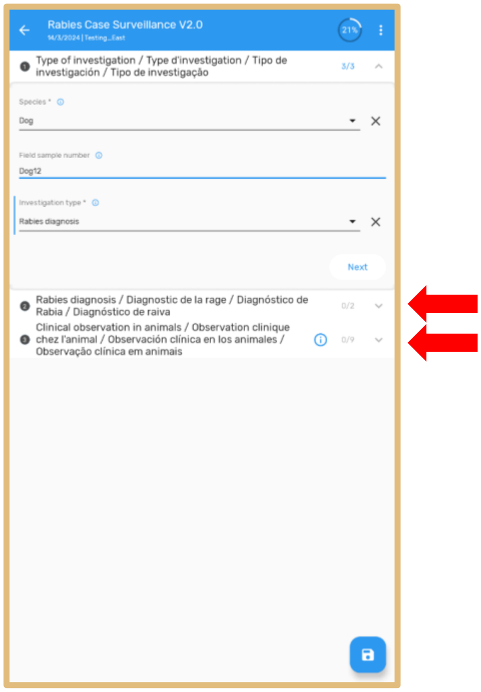

13. Open the next section of the form by clicking on the ‘Next’ icon.

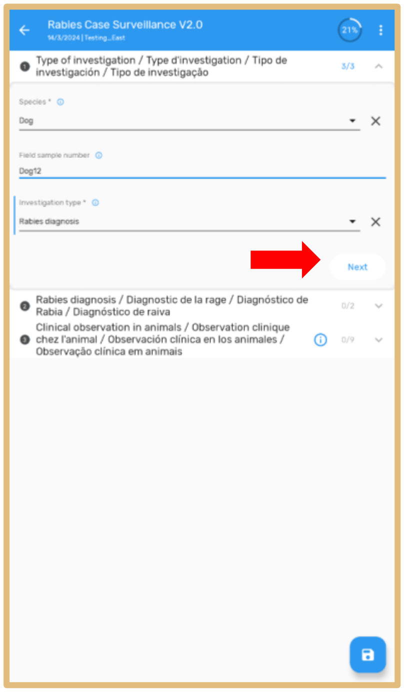

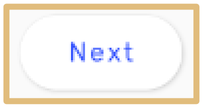

14. Select the diagnostic result.

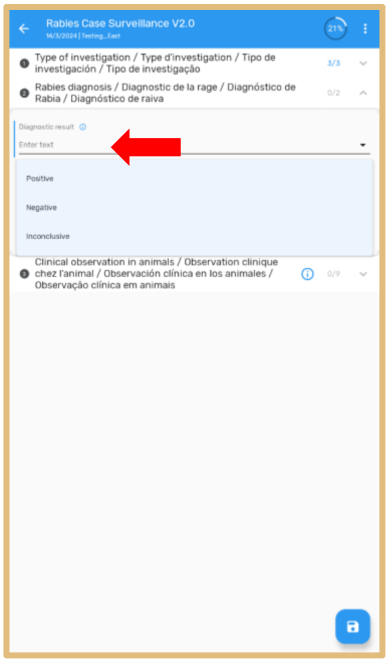

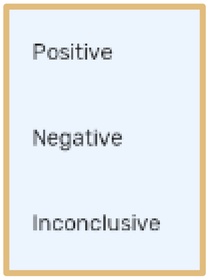

15. Enter the unique laboratory sample number. **NOTE**: If you are doing in-field diagnostic screening, leave the laboratory sample number field empty so that it can be completed after laboratory diagnosis.

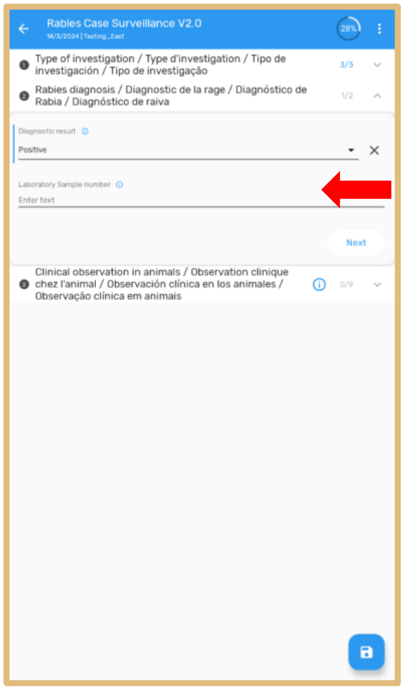

16. Open the next section of the form by clicking on the ‘Next’ icon.

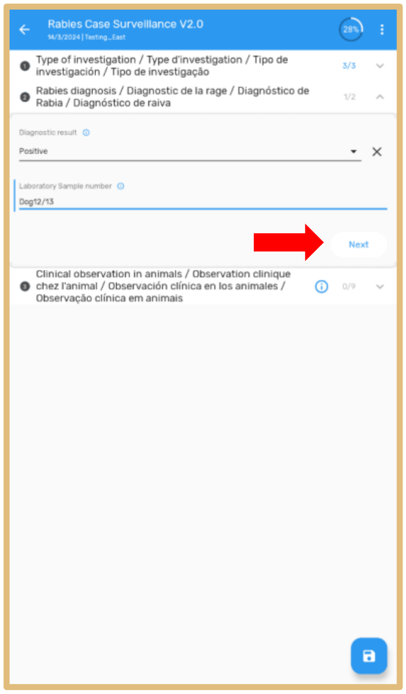

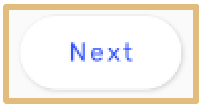

17. Additional data regarding the signs observed during the clinical observations of the animal can also be captured. Using the guide at the end of this document, select all the signs that were observed or reported. **If this information is not known, the section can be left blank.**

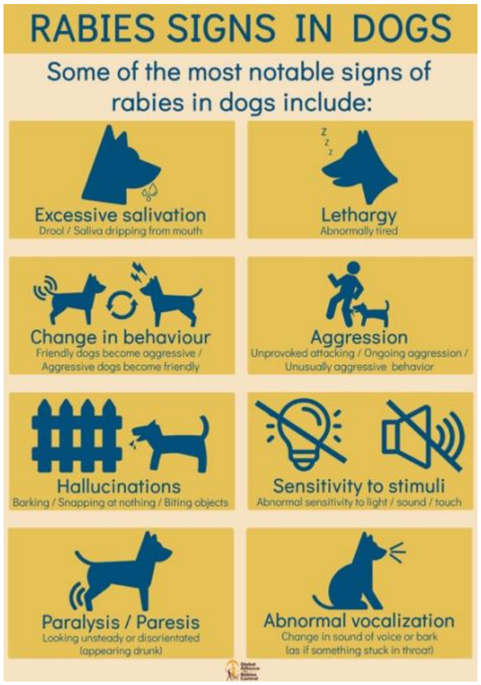

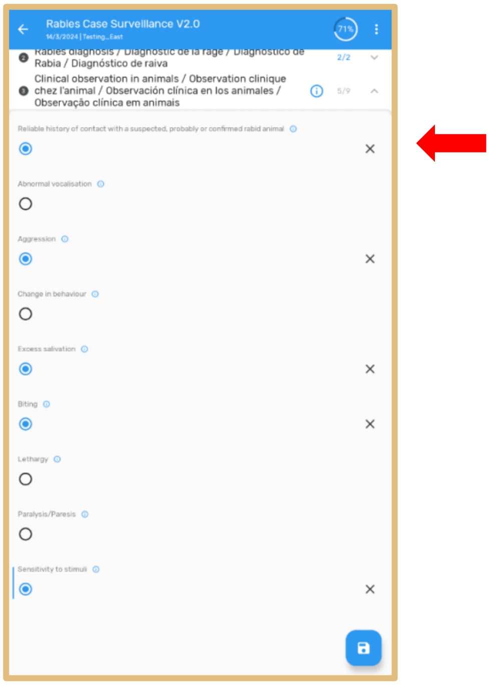

18. Click on the ‘Save’ button at the bottom right of the screen.

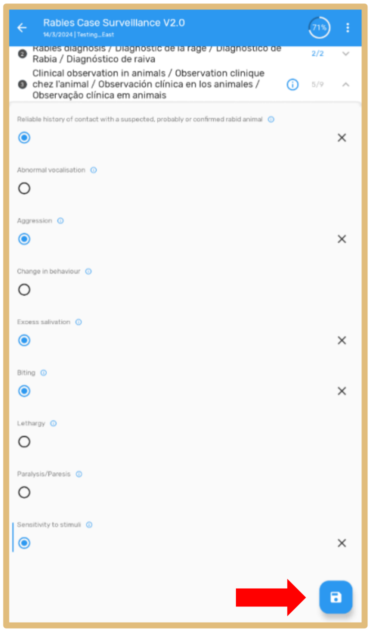

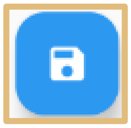

19. Select the appropriate option depending on your investigation.

NOTE:

- **Diagnostic Screening:** Selecting the ‘Not now’ button will leave the record active which can then easily be found and updated by laboratory technicians. **ONLY** select ‘Not now’ when you are doing diagnostic screening.
- **Laboratory confirmation:** When entering laboratory confirmation data, click on ‘Complete’.

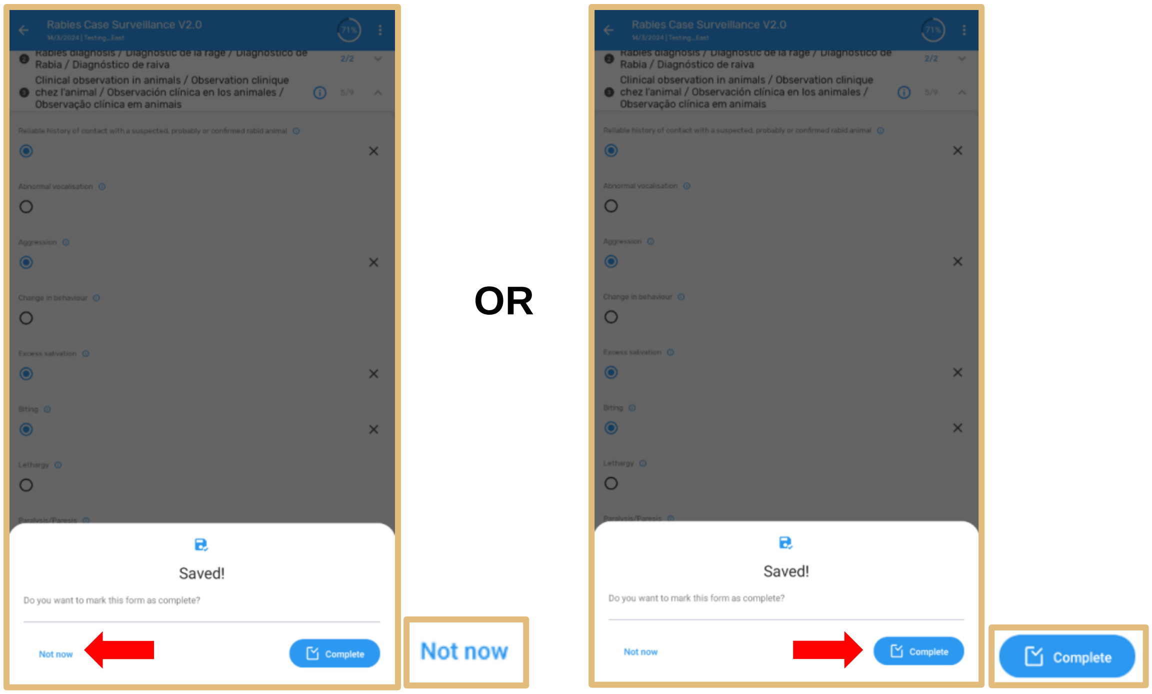

20. The rabies case is recorded on the GARC App and you can repeat this process for any other rabies cases. **NOTE**: Reported events that are active will be reflected on the app screen for the RCS tool. Note that completed events have green text with a check mark saying “Event completed”, whilst active events do not.

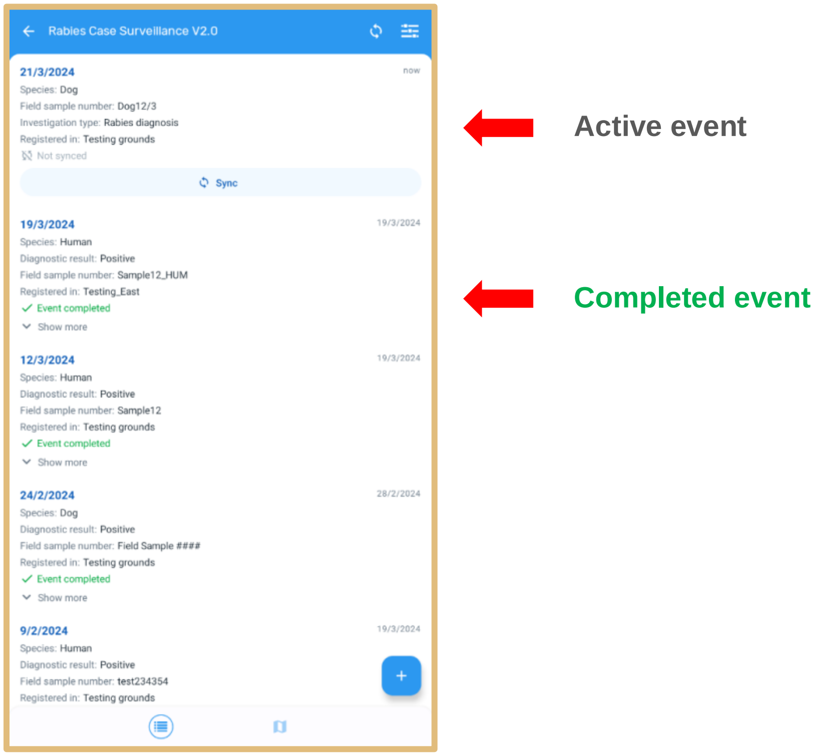

Capturing only clinical observation data

21. When selecting ‘Clinical observation’, only the section ‘Clinical observation in animals’ will open and allow you to capture observational data on the signs of rabies observed in an animal.

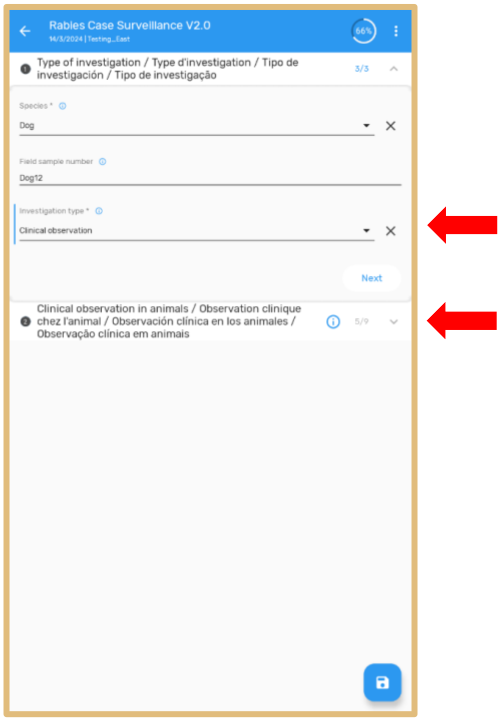

22. Open the next section of the form by clicking on the ‘Next’ icon.

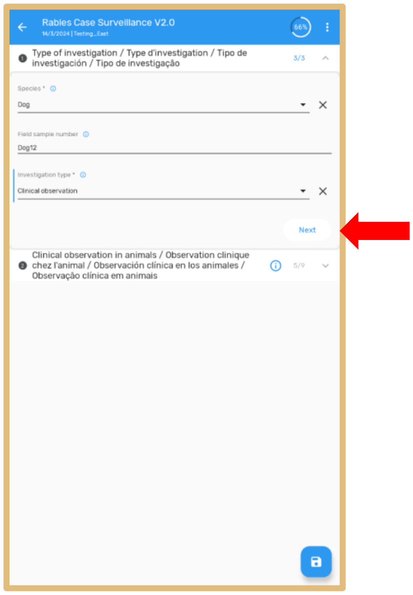

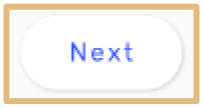

23. Using the guide at the end of the protocol, select all relevant signs of rabies observed in the animal.

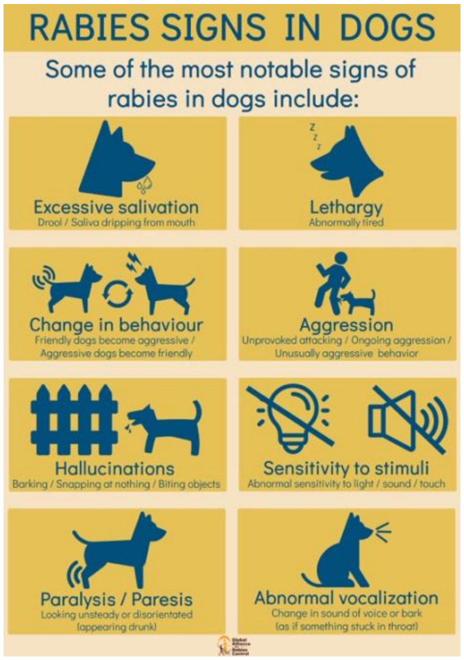

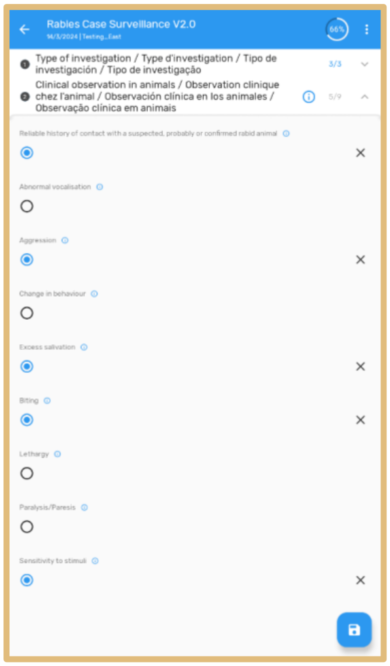

24. Click on the ‘Save’ button at the bottom right of the screen.

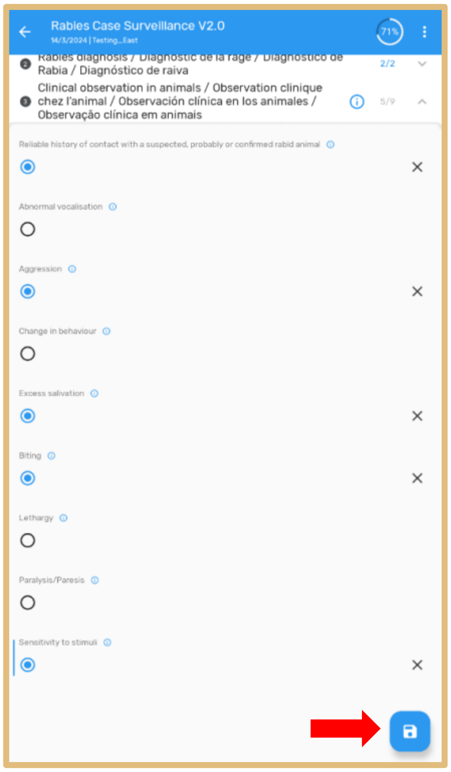

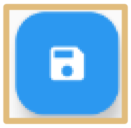

25. Select the appropriate option depending on your investigation. **NOTE**:

- **Diagnostic Screening:** Selecting the ‘Not now’ button will leave the record active which can then easily be found and updated by laboratory technicians. **ONLY** select ‘Not now’ when you are doing diagnostic screening.
- **Laboratory confirmation:** When entering laboratory confirmation data, click on ‘Complete’.

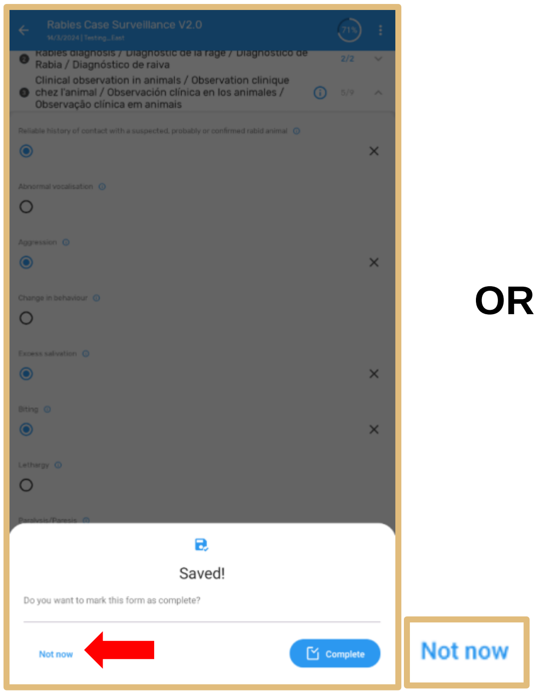

OR

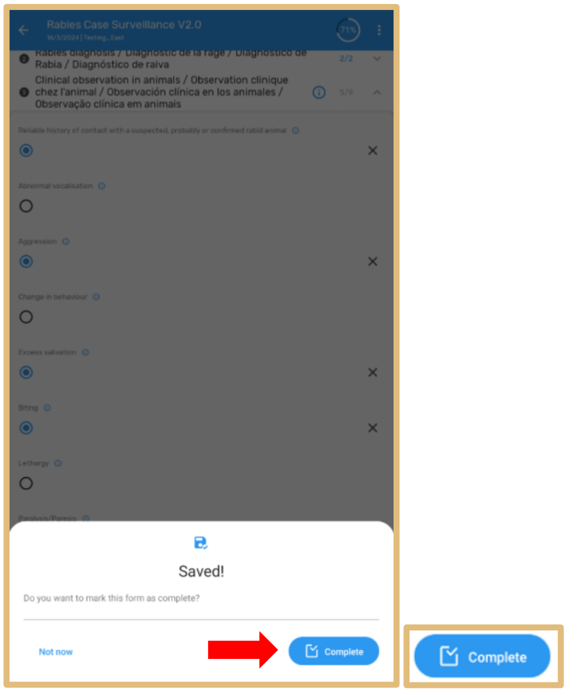

26. The rabies case is recorded on the GARC App and you can repeat this process for any other rabies cases.

Sending the case investigation data to the website

Important note

Syncing data is necessary to send all the data that has been stored on the device (done when working offline) to the Rabies Epidemiological Bulletin (REB). The REB then analyses the data and automatically creates or updates the maps and graphs on your dashboard. As such, data that has not been synced with the REB are stored on the device and will not display on the system's maps and graphs.

It is thus vital that you send the data to the REB as soon as possible. Ideally, each record captured should be synced with the REB immediately so that follow up investigations and interventions can be triggered.

Sending the data to the Rabies Epidemiological Bulletin

The grey 'circular arrows' symbol on the Home screen indicates that data on the GARC App has NOT been sent to the Rabies Epidemiological Bulletin. The data needs to be sent so that it can be included in the maps, graphs, and other visuals.

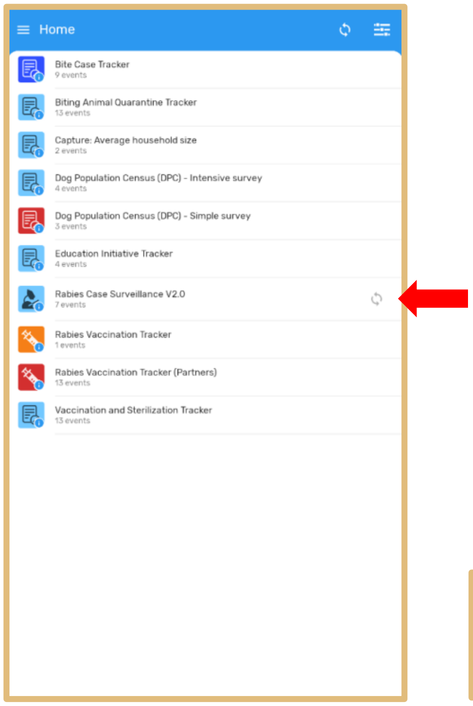

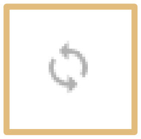

1. Click on the white 'circular arrows' symbol on to the 'Home' tab on the Home screen.

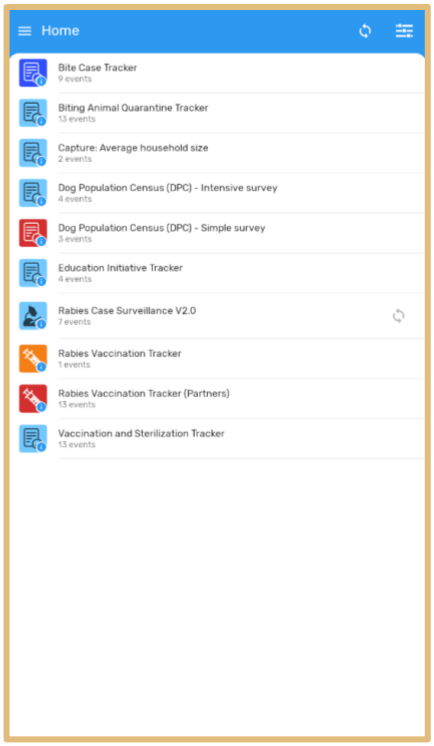

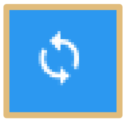

2. Ensure that the device is connected to the internet and click on the ‘Send’ button.

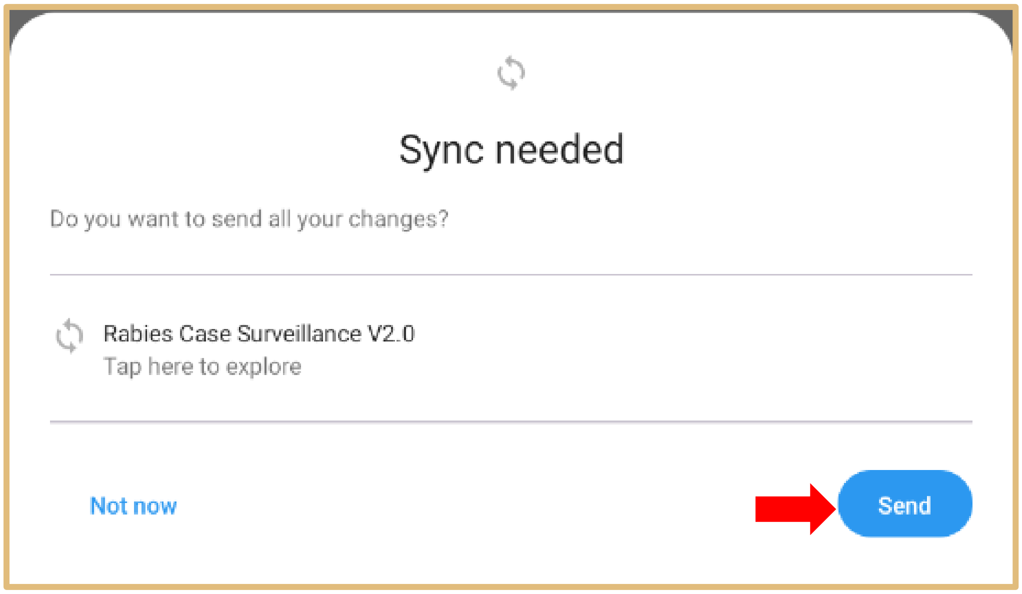

3. If successful, the following will appear:

.

a. The text will inform you that the sync was successful which shows data has been sent.
b. The grey arrows will disappear.

NOTE: All the data has now been sent to the website and the visuals (maps, graphs, and other visuals) will automatically update every hour, on the hour (GMT time). As such, additions or changes to the maps, graphs, and pivot tables will not appear instantly but will update after no more than 1 hour.

Step 4 – Sample storage and shipment to laboratory (protocol)

Overview:

All samples, regardless of the results obtained using ADTEC's "Rabies Ag test", must be sent to the nearest laboratory for diagnostic confirmation by means of one of the WOAH recommended techniques (DFA, DRIT, RT-PCR).

- Samples collected from a **biting animal** must be sent to the laboratory **as soon as possible** regardless of whether the animal tested positive, negative, or inconclusive for rabies.
- Samples that were collected from a **carcass that tested rabies-negative or inconclusive** need to be sent to the laboratory **as soon as possible**.
- Samples that were collected from a **carcass that tested rabies-positive** can be sent to the laboratory **when there is an opportunity to send a larger batch of samples at once** (i.e., once a week or once every two weeks).

Materials:

- Sample collection form
- Sealable, water-tight plastic bags
- Glycerol saline (500ml, prepared by mixing an equal volume of distilled glycerol and phosphate buffered saline (PBS)) (This should be prepared before going into the field).
- Box or container in which samples can be stored temporarily
- Packaging tape
- Marker/pen
- Packaging materials (including a secondary container and absorbent materials such as cotton wool or paper towel) in which sample storage tubes and LFDs can be sent to the laboratory.
- Nitrile/Latex gloves
- Bag for the last pair of gloves
- A bottle of clean water and liquid soap
- Disinfectant solution (10% bleach solution) in a spray bottle
- Paper towel

Methodology:

Packaging the sample for shipment

1. Put on a new pair of latex/nitrile gloves
2. Add glycerol saline to the sample collection tube - ensuring that the entire sample is completely covered.
3. Close the lid of the sample transport tube (containing the brain sample and glycerol saline) tightly and spray the outside lightly with disinfectant.
4. Leave sprayed bottle for 10 minutes in an uncontaminated area.
5. If the tube is not dry, dry off excess disinfectant with a piece of paper towel.
6. Place sample transport tube and the used test kit (see end of the LFD protocol) into a secondary shipping container that is ideally packed with absorbent material.
7. Spray outside of mailing container lightly with disinfectant and leave for 10 minutes in an uncontaminated area.
8. Wipe the plastic sheet clean of any **obvious** debris or biological materials (blood, hair, brain, etc.) with paper towel soaked in 10% bleach solution.
9. Spray down the entire plastic sheet work surface and throw it into the waste packet.
10. Discard any waste in the waste packet
11. Take off your personal protective equipment in an appropriate manner but **ensure that you leave your gloves on** so that you can place the personal protective equipment in the waste packet**.**
12. Spray down waste packet thoroughly and seal it by tying a tight knot.
13. Remove gloves and discard into the waste packet that is used for the last pair of gloves.
14. All waste packets should be incinerated at the end of the day.

Completing the paper-based form and finalizing the shipment

15. Complete the front page of the paper-based sample collection form for each sample (form attached to end of protocol).
16. Use the chart on the back page of the paper-based sample collection form to record the location where the sample was collected. **This location should be the same as the location that was selected in the mobile phone application** (form attached to end of protocol).
17. Place the paper-based sample collection form in a sealable, watertight container or bag. Store the sealed forms in the shipping container.
18. Seal the secondary shipping container (after it has air-dried) with tape. Ensure that you do this step **without** gloves as you want to prevent contamination of the outside of the container.
