# Supplementary material for: Investigating the Impact That Diagnostic Screening with Lateral Flow Devices Had on the Rabies Surveillance Program in Zanzibar, Tanzania
Source: Microorganisms. 2024 Jun 27;12(7):1314. doi: 10.3390/microorganisms12071314 (PMC11279036; doi:10.3390/microorganisms12071314)
Supplement: Supplementary file 1 [file microorganisms-12-01314-s001.zip › Table S1.pdf]

Table S1. Neuronal tissue sample cohort from Zanzibar depicting the diagnostic confirmation results using the DRIT assay at the Zanzibar Central Veterinary Laboratory.

#	Sample number	Date	Species	Location	District	DRIT result
1	010/16	21-07-2016	Canine	Kiongoni	South	Positive
2	011/16	27-07-2016	Caprine	Mkunazini	Urban	Positive
3	012/16	28-07-2016	Rodent	Rahaleo	Urban	Negative
4	013/16	03-08-2016	Bovine	Mwembe Makumbi	Urban	Negative
5	019/16	30-09-2016	Feline	Muembeshauri	Urban	Positive
6	020/16	13-10-2016	Feline	Amani	Urban	Positive
7	024/16	05-11-2016	Canine	Kidimni	Central	Positive
8	025/16	29-11-2016	Canine	Matetema	North B	Positive
9	026/16	17-12-2016	Feline	Fuoni Kijito Upele	West	Negative
10	001/17	06-01-2017	Equine	Ndijani mseweni	Central	Positive
11	002/17	09-01-2017	Feline	Bububu	West	Positive
12	003/17	17-01-2017	Canine	Mkadini	West	Positive
13	004/17	16-02-2017	Wildlife - Vervet monkey (*Chlorocebus pygerythrus*)	Charawe	South	Positive
14	008/17	28-02-2017	Wildlife - Vervet monkey (*Chlorocebus pygerythrus*)	Tumbatu	North A	Negative
15	009/17	28-02-2017	Wildlife - Vervet monkey (*Chlorocebus pygerythrus*)	Tumbatu	North A	Negative
16	010/17	28-02-2017	Wildlife - Vervet monkey (*Chlorocebus pygerythrus*)	Tumbatu	North A	Negative
17	011/17	28-02-2017	Wildlife - Vervet monkey (*Chlorocebus pygerythrus*)	Tumbatu	North A	Negative
18	012/17	28-02-2017	Wildlife - Vervet monkey (*Chlorocebus pygerythrus*)	Tumbatu	North A	Negative
19	013/17	02-03-2017	Caprine	Jambiani Kibigija	South	Positive
20	016/17	04-04-2017	Canine	Kajengwa	South	Positive
21	017/17	19-05-2017	Canine	Tomondo	West	Positive
22	018/17	19-05-2017	Feline	Makadara	Urban	Positive
23	027/17	09-06-2017	Caprine	Kajengwa	South	Positive
24	028/17	09-06-2017	Caprine	Kajengwa	South	Positive
25	029/17	29-06-2017	Feline	Amani	Urban	Positive
26	030/17	30-07-2017	Canine	Kidimni	Urban	Positive
27	031/17	05-08-2017	Feline	Kitope	North B	Positive
28	032/17	05-08-2017	Canine	Bububu	North B	Positive
29	033/17	10-08-2017	Feline	Mikunguni	Urban	Positive
30	034/17	10-08-2017	Canine	Kianga	West	Positive
31	039/17	10-08-2017	Caprine	Kombeni	West	Positive
32	040/17	10-08-2017	Caprine	Kombeni	West	Positive
33	041/17	04-12-2017	Canine	Kinyasini	North A	Positive
34	042/17	04-12-2017	Canine	Kinyasini	North A	Positive
35	043/17	04-12-2017	Caprine	Chuini	West	Positive
36	001/18	18-01-2018	Canine	Miwani	Central	Positive
37	002/18	28-02-2018	Canine	Cheju	Central	Positive
38	003/18	28-02-2018	Caprine	Maungani	West	Positive
39	004/18	23-04-2018	Caprine	Maungani	West	Positive
40	005/18	25-04-2018	Canine	Sharifumsa	West	Positive
41	006/18	28-06-2018	Canine	Kianga	West	Positive

42	007/18	12-07-2018	Canine	Mwera	West	Positive
43	008/18	31-07-2018	Canine	Mfenesini	West	Positive
44	001/19	08-01-2019	Wildlife - Red colobus monkey (*Piliocolobus kirkii*)	Pete	South	Positive
45	002/19	24-07-2017	Canine	Kisauni	West	Positive
46	003/19	21-11-2019	Canine	Bwejuu	South	Positive
47	004/19	14-12-2019	Canine	Kiwengwa	North B	Negative
48	005/19	16-12-2019	Bovine	Kizimbani	West	Positive
49	006/19	24-12-2019	Canine	Mkataleni	North B	Positive
50	007/19	24-12-2019	Canine	Michamvi	South	Positive
51	008/19	24-12-2019	Wildlife - Vervet monkey (*Chlorocebus pygerythrus*)	Bububu	North B	Positive
52	009/19	26-12-2019	Caprine	Mkataleni	North B	Positive
53	010/19	30-12-2019	Wildlife - Vervet monkey (*Chlorocebus pygerythrus*)	Jambiani Kibigija	South	Positive
54	001/20	23-01-2020	Canine	Jambiani Kibigija	South	Positive
55	002/20	18-02-2020	Caprine	Bububu	West	Positive
56	003/20	04-05-2020	Caprine	Bububu	West	Positive
57	004/20	02-07-2020	Feline	Fuoni Kibondeni	West	Positive
58	001/21	04-01-2021	Caprine	Mwera	West	Positive
59	002/21	21-01-2021	Wildlife - Vervet monkey (*Chlorocebus pygerythrus*)	Kiongoni	South	Positive
60	003/21	26-01-2021	Canine	Kigunda	North A	Positive
61	006/21	23-02-2021	Canine	Bububu	West	Positive
62	007/21	09-04-2021	Canine	Chwaka	Central	Negative
63	008/21	20-04-2021	Canine	Kianga	West	Positive
64	009/21	28-06-2021	Feline	Melinne	Urban	Negative
65	010/21	28-06-2021	Canine	Mikunguni	Urban	Negative
66	011/21	25-07-2021	Caprine	Bububu	West	Negative
67	012/21	26-11-2021	Canine	Mkwajuni	West	Positive
