# Supplementary material for: Investigating the Impact That Diagnostic Screening with Lateral Flow Devices Had on the Rabies Surveillance Program in Zanzibar, Tanzania
Source: Microorganisms. 2024 Jun 27;12(7):1314. doi: 10.3390/microorganisms12071314 (PMC11279036; doi:10.3390/microorganisms12071314)
Supplement: Supplementary file 1 [file microorganisms-12-01314-s001.zip › Table S2.pdf]

Table S2. Neuronal tissue sample cohort from Zanzibar depicting the initial in-field diagnostic screening results and their diagnostic confirmation using the DRIT assay at the Zanzibar Central Veterinary Laboratory.

#	Sample number	Date	Species	Location	District	LFD result	DRIT result	Reason for diagnostic screening
1	001/22	10-03-2022	Canine	Pongwe Pwani	Central	Positive	Positive	Animal was showing signs of rabies and was humanely euthanised.
2	002/22	14-03-2022	Feline	Kilimani	Urban	Negative	Negative	Roadkill
3	003/22	20-04-2022	Feline	Kianga	West	Negative	Negative	Roadkill
4	004/22	21-04-2022	Feline	Amani	Urban	Negative	Negative	Roadkill
5	005/22	28-04-2022	Canine	Dunga Kiembeni	Central	Negative	Negative	Animal was showing signs of rabies and was humanely euthanised.
6	006/22	28-04-2022	Wildlife - Red colobus monkey (*Piliocolobus kirkii*)	Pete	South	Negative	Negative	Animal was showing signs of rabies and was humanely euthanised.
7	007/22	01-05-2022	Feline	Mpendae	Urban	Negative	Negative	Roadkill
8	008/22	05-05-2022	Canine	Chukwani	West	Negative	Negative	Animal was showing signs of rabies and was humanely euthanised.
9	009/12	25-05-2022	Feline	Mikunguni	Urban	Negative	Negative	Animal was showing signs of rabies and was humanely euthanised.
10	010/22	27-05-2022	Canine	Ndijani mseweni	Central	Positive	Positive	Animal was showing signs of rabies and was killed by community members.
11	011/22 *	12-06-2022	Canine	Ndijani mseweni	Central	Negative	Positive	Animal was showing signs of rabies and was killed by community members.
12	012/22	14-06-2022	Feline	Gulioni	Urban	Negative	Negative	Roadkill
13	013/22	17-06-2022	Canine	Dunga Bweni	Central	Negative	Negative	Animal was showing signs of rabies and was humanely euthanised.
14	014/22	28-06-2022	Canine	Jambiani Kibigija	South	Negative	Negative	Roadkill
15	015/22	05-07-2022	Feline	Mikunguni	Urban	Negative	Negative	Animal was showing signs of rabies and was humanely euthanised.
16	016/22	05-07-2022	Canine	Dole	West	Negative	Negative	Roadkill
17	017/22	06-07-2022	Feline	Paje	South	Negative	Negative	Animal was showing signs of

								rabies and was humanely euthanised.
18	018/22	18-07-2022	Canine	Pagali	Central	Negative	Negative	Animal was showing signs of rabies and was killed by community members.
19	019/22	23-08-2022	Canine	Fuoni Migombani	West	Negative	Negative	Animal was showing signs of rabies and was killed by community members.
20	020/22	01-09-2022	Canine	Kibweni	West	Positive	Positive	Animal was showing signs of rabies and was humanely euthanised.
21	021/22	15-09-2022	Canine	Maungani	West	Positive	Positive	Animal was showing signs of rabies and was humanely euthanised.
22	022/22	04-10-2022	Feline	Mwanakwerekwe	West	Negative	Negative	Roadkill
23	023/22	12-10-2022	Feline	Muembeshauri	Urban	Negative	Negative	Roadkill
24	024/22	12-10-2022	Caprine	Nyamanzi	West	Negative	Negative	Animal was showing signs of rabies and was killed by community members.
25	025/22	24-10-2022	Bat	Dunga Bweni	Central	Negative	Negative	Found dead
26	026/22	28-10-2022	Canine	Uzini	Central	Negative	Negative	Animal was showing signs of rabies and was humanely euthanised.
27	027/22	28-10-2022	Canine	Uzini	Central	Negative	Negative	Animal was showing signs of rabies and was humanely euthanised.
28	028/22	02-11-2022	Canine	Bububu	West	Negative	Negative	Roadkill
29	029/22	03-11-2022	Canine	Ndijani mseweni	Central	Positive	Positive	Animal was showing signs of rabies and was humanely euthanised.
30	030/22	23-11-2022	Feline	Kikwajuni Bondeni	Urban	Positive	Positive	Animal was showing signs of rabies and was humanely euthanised.
31	031/22	04-12-2022	Canine	Mwembe Makumbi	Urban	Negative	Negative	Roadkill
32	032/22	04-12-2022	Canine	Fuoni Kibondeni	West	Negative	Negative	Animal was showing signs of rabies and was humanely euthanised.
33	033/22	24-12-2022	Canine	Ndijani mseweni	Central	Positive	Positive	Animal was showing signs of rabies and was humanely euthanised.

34	034/22	28-12-2022	Canine	Maungani	West	Negative	Negative	Animal was showing signs of rabies and was humanely euthanised.
35	035/22	28-12-2022	Canine	Bungi	Central	Negative	Negative	Animal was showing signs of rabies and was humanely euthanised.
36	036/22	28-12-2022	Canine	Nungwi	North A	Negative	Negative	Animal was showing signs of rabies and was humanely euthanised.
37	001/23	09-01-2023	Canine	Maungani	West	Negative	Negative	Animal was showing signs of rabies and was humanely euthanised.
38	002/23	11-01-2023	Wildlife - Red colobus monkey (*Piliocolobus kirkii*)	Jozani	South	Negative	Negative	Roadkill
39	003/23	12-01-2023	Canine	Pagali	Central	Negative	Negative	Animal was showing signs of rabies and was killed by community members.
40	004/23	19-01-2023	Canine	Jambiani Kibigija	South	Negative	Negative	Animal was showing signs of rabies and was humanely euthanised.
41	006/23	25-01-2023	Canine	Tunguu	Central	Positive	Positive	Animal was showing signs of rabies and was humanely euthanised.
42	007/23	31-01-2023	Canine	Kisauni	West	Negative	Negative	Animal was showing signs of rabies and was humanely euthanised.
43	008/23	01-02-2023	Bovine	Kilombero	North B	Positive	Positive	Animal was showing signs of rabies and was killed by community members.
44	009/23	10-02-2023	Canine	Kiwengwa	North B	Positive	Positive	Animal was showing signs of rabies and was humanely euthanised.
45	010/23	11-02-2023	Canine	Bububu	West	Positive	Positive	Animal was showing signs of rabies and was humanely euthanised.
46	011/23	14-02-2023	Canine	Kisauni	West	Negative	Negative	Animal was showing signs of rabies and was humanely euthanised.
47	012/23	14-02-2023	Canine	Fuoni Kibondeni	West	Positive	Positive	Animal was showing signs of rabies and was

								humanely euthanised.
48	013/23	16-02-2023	Canine	Maungani	West	Negative	Negative	Animal was showing signs of rabies and was humanely euthanised.
49	014/23	21-02-2023	Canine	Jambiani kikadini	South	Negative	Negative	Animal was showing signs of rabies and was humanely euthanised.
50	015/23	20-03-2023	Feline	Kama	West	Negative	Negative	Animal was showing signs of rabies and was humanely euthanised.
51	016/23	21-03-2023	Canine	Kiembesamaki	West	Positive	Positive	Animal was showing signs of rabies and was humanely euthanised.
52	017/23	26-03-2023	Canine	Kilombero	North B	Positive	Positive	Animal was showing signs of rabies and was humanely euthanised.
53	018/23	28-03-2023	Canine	Tunguu	Central	Positive	Positive	Animal was showing signs of rabies and was humanely euthanised.
54	019/23	01-04-2023	Rodent	Kizimbani	West	Negative	Negative	Found dead
55	020/23	03-04-2023	Canine	Kiembesamaki	West	Positive	Positive	Animal was showing signs of rabies and was humanely euthanised.
56	021/23	13-04-2023	Canine	Kitogani	South	Negative	Negative	Roadkill
57	022/23	04-05-2023	Feline	Kisiwandui	Urban	Negative	Negative	Animal was showing signs of rabies and was humanely euthanised.
58	023/23	05-05-2023	Canine	Pete	South	Negative	Negative	Roadkill
59	024/23	15-05-2023	Canine	Fuoni Kipungani	West	Negative	Negative	Animal was showing signs of rabies and was humanely euthanised.
60	025/23	19-05-2023	Feline	Shangani	Urban	Negative	Negative	Animal was showing signs of rabies and was humanely euthanised.
61	026/23	22-05-2023	Canine	Pangawe	West	Negative	Negative	Animal was showing signs of rabies and was humanely euthanised.
62	027/23	24-05-2023	Feline	Mwanakwerekwe	West	Negative	Negative	Animal was showing signs of rabies and was humanely euthanised.
63	028/23	08-06-2023	Feline	Kilombero	North B	Positive	Positive	Animal was showing signs of rabies and was

								humanely euthanised.
64	029/23	18-06-2023	Feline	Paje	South	Negative	Negative	Animal was showing signs of rabies and was humanely euthanised.
65	030/23	18-06-2023	Canine	Dole	West	Negative	Negative	Animal was showing signs of rabies and was humanely euthanised.
66	031/23	18-06-2023	Canine	Dole	West	Negative	Negative	Animal was showing signs of rabies and was humanely euthanised.
67	032/23	21-06-2023	Wildlife - Red colobus monkey (*Piliocolobus kirkii*)	Dole	West	Negative	Negative	Animal was showing signs of rabies and was humanely euthanised.
68	033/23	26-06-2023	Ovine	Kibweni	West	Negative	Negative	Animal was showing signs of rabies and was humanely euthanised.
69	034/23	27-06-2023	Feline	Mombasa	West	Negative	Negative	Animal was showing signs of rabies and was humanely euthanised.
70	035/23	16-07-2023	Ovine	Kizimbani	West	Negative	Negative	Animal was showing signs of rabies and was humanely euthanised.
71	036/23	25-07-2023	Wildlife - Bush Baby (*Paragalago zanzibaricus*)	Chuini	West	Negative	Negative	Animal was showing signs of rabies and was humanely euthanised.
72	037/23	29-07-2023	Feline	Fukuchani	North A	Negative	Negative	Animal was showing signs of rabies and was humanely euthanised.
73	038/23	29-07-2023	Feline	Mtoni	West	Negative	Negative	Animal was showing signs of rabies and was humanely euthanised.
74	039/23	30-07-2023	Feline	Kiponda	Urban	Negative	Negative	Animal was showing signs of rabies and was humanely euthanised.
75	040/23	29-08-2023	Canine	Mtoni kidatu	West	Negative	Negative	Found dead
76	041/23	29-08-2023	Canine	Mtoni kidatu	West	Negative	Negative	Found dead
77	042/23	29-08-2023	Canine	Mtoni kidatu	West	Negative	Negative	Found dead
78	043/23	17-10-2023	Wildlife – African Civet (*Nandinia binotata*)	Cheju	Central	Negative	Negative	Roadkill
79	044/23	25-10-2023	Canine	Dole	West	Negative	Negative	Roadkill
80	045/23	03-11-2023	Canine	Mwembe Makumbi	Urban	Negative	Negative	Animal was showing signs of rabies and was

								humanely euthanised.
81	046/23	09-11-2023	Feline	Tindini	Central	Positive	Positive	Animal was showing signs of rabies and was humanely euthanised.
82	047/23	14-11-2023	Canine	Tindini	Central	Positive	Positive	Animal was showing signs of rabies and was humanely euthanised.
83	048/23	03-12-2023	Canine	Nungwi	North A	Negative	Negative	Animal was showing signs of rabies and was humanely euthanised.

* Denotes samples that produced inconsistent results with the LFD and DRIT assay.
